# Memory-like CD8^+^ T cells lacking PD-1 adapt to persistent stimulation by reducing TCR signal transduction rather than increasing exhaustion

**DOI:** 10.3389/fimmu.2026.1743170

**Published:** 2026-02-05

**Authors:** Mélanie Charmoy, Julia M. Maier, Tania Wyss, Vijaykumar Chennupati, Catherine Sabatel, Laurie Bonneaux, Alexandre Dumez, Romain Veber, Greta Guarda, Werner Held

**Affiliations:** 1 Department of Fundamental Oncology, University of Lausanne, Lausanne, Switzerland; 2 Translational Data Science Facility, AGORA Cancer Research Center, Swiss Institute of Bioinformatics., Lausanne, Switzerland; 3 Institute for Research in Biomedicine, Università della Svizzera Italiana, Bellinzona, Switzerland

**Keywords:** CD8 T cells, chronic viral infection, exhaustion, lymphocytic choriomeningitis virus (LCMV), stemness

## Abstract

CD8^+^ T cells respond to persistent stimulation during chronic viral infection by stably expressing co-inhibitory receptors and other exhaustion-related molecules. Here we addressed how memory-like CD8^+^ T (T_ML_) cells, which sustain the immune response to chronic infection thanks to their stem-like properties, adapt to chronic stimulation when they cannot express the co-inhibitory receptor PD-1. We found an increased initial generation and stable long-term persistence of T_ML_ cells lacking PD-1 during chronic viral infection. However, these cells had a reduced ability regenerate upon acute restimulation in the context of a recall response. Mechanistically, the lack of PD-1-mediated inhibition was not compensated by an increased expression of other co-inhibitory receptors or exhaustion related molecules. Rather, the absence of PD-1 resulted in a reduced capacity of the TCR to activate T_ML_ cells and to express stemness genes including *Myb* and *Klf4*. Similar albeit weaker effects on T_ML_ cells were noted when PD-1 engagement was transiently interrupted due to anti-PD-L1 treatment. Thus, stem-like CD8^+^ T cells responding to chronic viral infection adapt to the absence of PD-1-dependent co-inhibitory signals by further reducing TCR-mediated activation signaling, likely to prevent excessive or prolonged stimulation of these cells.

## Introduction

Persistent activation reduces the capacity of CD8^+^ T cells to proliferate and exert effector functions, including cell-mediated killing and production of IFNγ and TNFα. A hallmark of these so-called exhausted CD8^+^ T cells is the upregulation of co-inhibitory receptors including PD-1, Lag3 and Tim3 (1, 2). PD-1 plays a critical and non-redundant role in protecting mice against excessive CD8^+^ T cell-mediated immunopathology (3, 4). PD-1 expression is induced in response to T cell activation (5) and is constitutively expressed when T cell receptor (TCR) stimulation persists (6). Engagement of PD-1 by its ligands PD-L1 or PD-L2 results in the recruitment of phosphatases, including SHP2, to the cytoplasmic tail and this counteracts positive signaling events triggered by the TCR including the phosphorylation of CD3z, ZAP-70, PKCθ, PI3K, RAS and CD28 (7–9), which are essential for proper T cell activation, proliferation and effector responses. Indeed, interrupting PD-1/PD-L1 interaction during chronic viral infection, is sufficient to increase TCR signaling (as judged by *Nr4a1* upregulation), to improve the lysis of antigen-bearing target cells, the production of cytokines and to mediate a limited expansion of virus-specific CD8^+^ T cells *in vivo* (3, 10), demonstrating that PD-1 provides central and non-redundant inhibitory signals to CD8^+^ T cells persistently exposed to antigen. In addition, chronic stimulation alters TCR downstream signaling. NFATc1 is transcriptionally upregulated, whereas Fos, which is part of the AP-1 dimer, is downregulated (11). In the relative absence of AP-1, partner-less NFAT binds regulatory regions of coinhibitory receptor genes, including *Pdcd1* (PD-1) and *Havcr2* (Tim3) (12). Persistent NFAT activation further upregulates TOX, which orchestrates the upregulation of co-inhibitory receptors and thus the exhaustion program and ensures the survival of chronically stimulated T cells (13-15).

Whether and how CD8^+^ T cells cope with chronic stimulation when they cannot express the co-inhibitory receptor PD-1 has remained incompletely understood. Wild-type recipient mice adoptively transferred with a high dose of virus-specific PD-1 KO CD8^+^ T cells die around day 10 post infection with an LCMV strain causing chronic infection, while mice receiving a low dose survive long-term. In the latter case, virus-specific PD-1 KO CD8^+^ T cells expand considerably more than wild type and are maintained at higher numbers for an extended period of time. However, their presence does not significantly reduce viremia or increase morbidity during chronic viral infection (16), suggesting that these cells do not exhibit increased functionality. These observations raise the question of how CD8^+^ T cells adapt to chronic stimulation when they cannot express PD-1.

Studies addressing the impact of PD-1 deficiency have investigated effects on the entire population of virus-specific CD8^+^ T cells (16). However, more recent studies revealed that virus-specific CD8^+^ T cells are heterogenous and that a subpopulation, so called memory-like CD8^+^ T (T_ML_) cells (also termed progenitor exhausted T cells), which have stem cell-like properties, are essential to sustain the CD8^+^ T cells response to chronic infection (17, 18). T_ML_ cells, which display features of central memory cells (Tcf1, Id3, CD62L, CCR7) combined with that of exhausted cells (Tox, PD-1, Lag-3*)*, continuously produce more differentiated or exhausted (T_EX_) cells, that lack Tcf1 but acquire cytolytic effector potential (GzmB) and additional inhibitory receptors including Tim3. T_ML_ stemness is also evident based on their capacity to expand and self-renew or differentiate in response to inhibitory receptor blockade or recall stimulation (17, 18). These findings raise the additional question regarding the importance of PD-1 in the generation, maintenance and/or function of T_ML_ cells.

Here we show an increased initial generation and stable long-term persistence of T_ML_ cells lacking PD-1 during chronic viral infection. Although their maintenance upon retransfer into chronically infected mice was normal, PD-1 KO T_ML_ cells had a reduced capacity to regenerate in response to acute recall stimulation. The absence of PD-1 was not compensated by an increased expression of other inhibitory receptors or exhaustion related genes. Rather, the TCR of PD-1 KO T_ML_ cells had a reduced capacity to activate these cells. Corresponding although weaker effects were noted when T_ML_ cells derived from chronically infected mice that had been subjected to PD-L1 blockade. Thus, PD-1 preserves the ability of T_ML_ cells to regenerate in response to rechallenge.

## Results

### Increased generation and relatively stable long-term persistence of PD-1 KO T_ML_ cells during chronic viral infection

**Figure 1:**
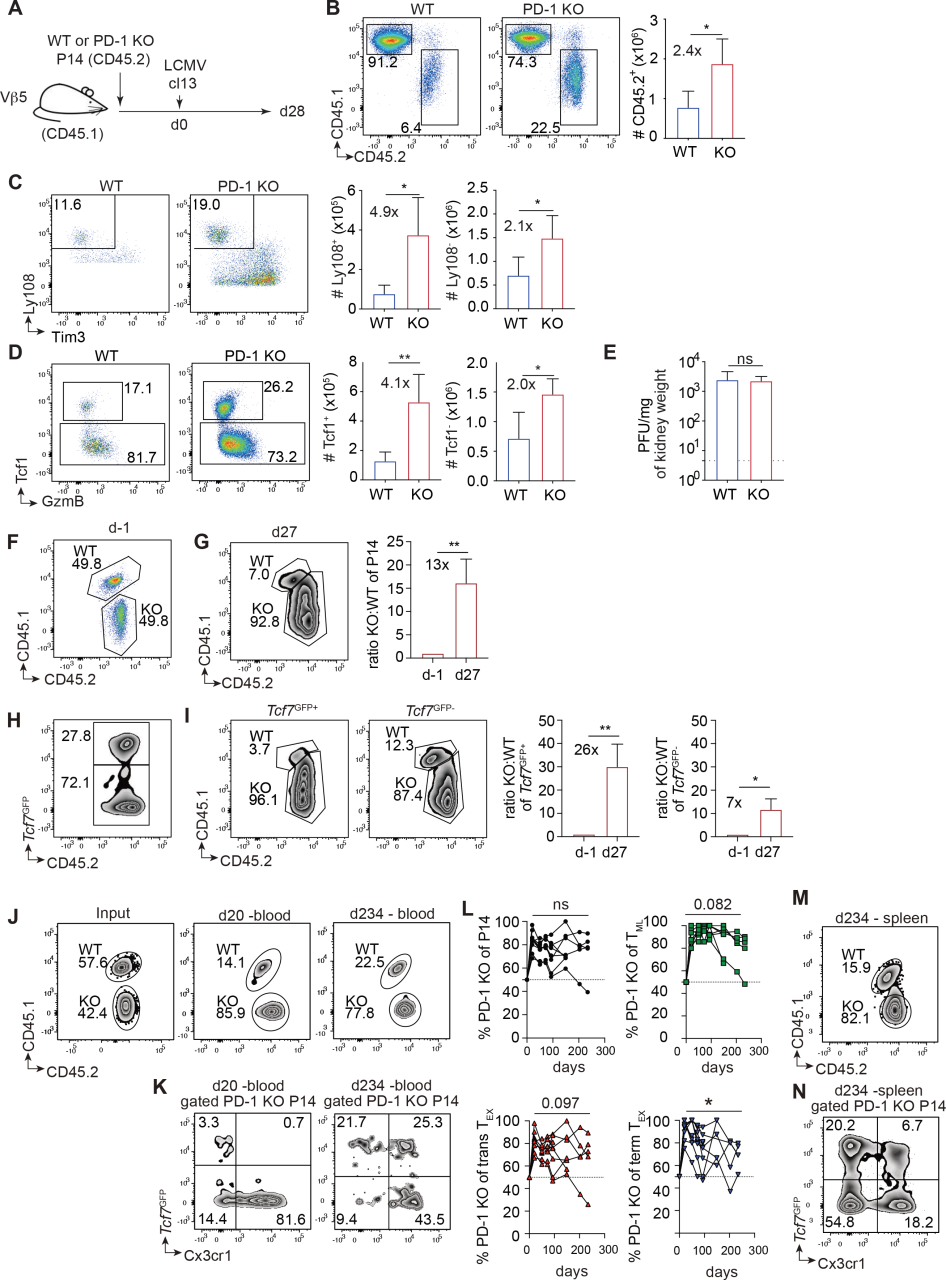
Increased generation and stable persistence of PD-1 KO T_ML_ cells during chronic viral infection. **(A–D)** Naïve WT or PD-1 KO P14 cells (CD45.2) (500 cells), were injected into V*β*5 mice (CD45.1), which were then infected with LCMV cl13 and analyzed at d28 post infection (p.i.). **(B)** Abundance of WT or PD-1 KO P14 cells (CD45.2^+^) among gated CD8^+^ T cells in the spleen of chronically infected mice at d28 p.i. **(C, D)** Gated P14 cells were analyzed for the presence of **(C)** Ly108^+^ Tim3^-^ T_ML_ versus Ly108^-^ T_EX_ cells or **(D)** Tcf1^+^ T_ML_ vs Tcf1^-^ GzmB^+^ T_EX_ cells. **(E)** Virus titers (plaque forming units (PFU)) in the kidney at d28 p.i. **(F–I)** PD-1 KO (CD45.2) and WT *Tcf7*^GFP+^ P14 (CD45.1/2) cells were mixed (250 cells each) prior to transfer into Vβ5 mice (CD45.1) and infection LCMV cl13. **(G)** The spleens of mice were analyzed 27 days later and gated P14 cells were analyzed for the contribution of PD-1 KO cells (CD45.2). The bar graph indicates the ratio of PD-1 KO to WT cells **(H)**. P14 cells were analyzed for *Tcf7*^GFP^ expression and **(I)** gated *Tcf7*^GFP+^ (T_ML_) and *Tcf7*^GFP-^ P14 cells (T_EX_) were analyzed for the contribution of PD-1 KO cells. **(J–N)** PD-1 KO (CD45.2) and WT *Tcf7*^GFP^ P14 (CD45.1/2) cells were mixed (Input: 42.4% KO) prior to transfer into Vβ5 mice (CD45.1) and infection with LCMV cl13. **(J)** At the indicated time points p.i, gated WT and PD-1 KO P14 cells present in the blood were analyzed for **(K)***Tcf7*^GFP^ versus Cx3Cr1 expression. **(L)** Gated *Tcf7*^GFP+^ T_ML_ and *Tcf7*^GFP-^ Cx3cr1^+^ trans T_EX_ or *Tcf7*^GFP-^ Cx3cr1^-^ term T_EX_ P14 cells were analyzed for the contribution of PD-1 KO cells. **(M)** Gated PD-1 KO P14 cells present in the spleen at d234 p.i. were analyzed for **(N)***Tcf7*^GFP^ versus Cx3Cr1 expression. Data in **(B–E)** derive from 4–5 mice per group and are representative of at least n=4 independent experiments. Data in **(F–I)** derive from 4–5 mice per group and are representative of n=3–4 independent experiments. Data in **(J–N)** derive from 5–10 mice time point. Bar graphs show means (± SD). Statistics: **(B–E)** Unpaired two-tailed t-test (between WT versus PD-1KO subsets). **(F–I)** Paired two-tailed t-test. **(J–N)** Paired t-test was used to determine statistically significant differences in the contribution of PD-1 KO cells between the d20 and d234 p.i. Significance (*p<0.05, **p<0.01, ns=not significant p>0.05).

To address the impact of PD-1-deficiency on chronically stimulated memory-like CD8^+^ T (T_ML_) cells we transferred wild type (WT) or *Pdcd1^-/-^* (PD-1 KO) CD8^+^ T cells expressing a TCR specific for the LCMV epitope gp33-41 (P14 cells) (CD45.2) into TCRβ (Vβ5) transgenic mice (CD45.1), followed by infection with LCMV clone 13 (cl13), which causes chronic infection (**Figure 1A**, **Supplementary Figure S1A**). Vβ5 mice show a reduced endogenous T cell response to LCMV and, similar to CD4-depleted B6 mice, have higher LCMV cl13 titers than C57BL/6 mice (6). Vβ5 mice adoptively transferred with 5000 PD-1 KO P14 cells died around d12 post infection (p.i.), but survived when transferred with 5000 WT P14 cells or with 500 PD-1 KO P14 cells. Gated P14 cells present in the spleen were analyzed on d8 (**Supplementary Figure S1B**) or d28 p.i. (**Figures 1A-F**).

At the acute phase of LCMV cl13 infection (d8 p.i.), PD-1 KO P14 cells had expanded around 6-fold more than WT P14 cells, with corresponding increases among PD-1 KO T_ML_ (Ly108^+^ Tim3^-^ or Tcf1^+^ GzmB^-^) and T_EX_ cells (Ly108^-^ or Tcf1^-^ GzmB^+^) (**Supplementary Figure S1B**). At d28 p.i., recipient spleens harbored 2.4-fold more PD-1 KO compared to WT P14 cells (**Figure 1B**), in agreement with (16). The abundance of PD-1 KO T_ML_ cells was increased 4-fold, while that of PD-1 KO T_EX_ cells was increased 2-fold (**Figures 1C, D**). Despite the increased abundance of PD-1 KO P14 cells, virus titers in the kidney were not different compared to mice transferred with WT P14 cells (**Figure 1E**), indicating that chronic phase PD-1 KO cells were not more effective in suppressing virus infection than WT cells. PD-1 KO T_ML_ cells showed enhanced cycling and comparable survival on d8 but comparable cycling and reduced survival on d28 p.i. (**Supplementary Figures S1C, D**), explaining the increased initial generation of PD-1 KO T_ML_ cells and suggesting that their long-term survival may be reduced.

To compare the fitness of WT and PD-1 KO T_ML_ cells in the same infectious and inflammatory environment, we transferred a mix of naïve WT P14 (CD45.1/2) and PD-1 KO P14 cells (CD45.2) (49.8% KO cells) (both expressing a *Tcf7*^GFP^ reporter) into Vβ5 recipients (CD45.1) (**Figure 1F**, **Supplementary Figures S2A, B**). At d8 p.i. the mix was skewed around 4-fold in favor of PD-1 KO P14 cells with corresponding skews for PD-1 KO T_ML_ (*Tcf7*^GFP+^) and T_EX_ cells (*Tcf7*^GFP-^) (**Supplementary Figures S2C–E**). At d28 p.i., the mix was skewed further in favor of PD-1 KO P14 cells (13-fold) whereby PD-1 KO T_ML_ and T_EX_ cells (*Tcf7*^GFP-^) were 26-fold and 7-fold more abundant, respectively, than the corresponding WT subpopulations (**Figures 1G–I**). Thus, PD-1 KO T_ML_ cells were considerably more competitive than WT T_ML_ cells.

To see whether the increased competitiveness of PD-1 KO T_ML_ cells was durable, we followed the relative presence of WT and PD-1 KO cells in the blood of chronically infected mice over an extended period of time. In agreement with the analyses of the spleen, d20 p.i. blood borne PD-1 KO T_ML_ cells were considerably skewed towards KO T_ML_ cells (85.9% KO) compared to input (42.4% KO) (**Figure 1J**). The elevated contribution of PD-1 KO cells remained stable until d234 p.i, although occasional mice showed a drop at late stages (**Figure 1J, L**). Separating P14 cells into T_ML_ (*Tcf7*^GFP+^Cx3cr1^-^), transitory T_EX_ (trans T_EX_: *Tcf7*^GFP-^Cx3cr1^+^) and terminal T_EX_ (term T_EX_: *Tcf7*^GFP-^Cx3cr1^-^) subsets (19) revealed a stably elevated contribution of PD-1 KO cells to the T_ML_ and the trans T_EX_ but a reduced contribution to the term T_EX_ compartment (**Figure 1K**). The observations in the blood were confirmed in the spleen upon sacrifice at d234 p.i. (**Figure 1M, N**). The phenotypic analysis revealed the emergence of P14 cells with a *Tcf7*^GFP+^Cx3cr1^+^ phenotype around d100 p.i. that gradually expanded over time (**Figures 1J–N**). This new subset was observed both in the blood and the spleen among both WT and PD-1 KO P14 cells, and was skewed towards PD-1 KO cells similar to T_ML_ cells. Thus, following an increased generation, PD-1 KO T_ML_ cells were maintained at high levels for extended periods of time although their ability to generate term T_EX_ cells started to decline at late stages of the infection.

### Chronic phase PD-1 KO T_ML_ cells have reduced self-renewal capacity in response to recall stimulation

The increased presence of PD-1 KO T_ML_ cells prompted us to compare their stemness to that of WT T_ML_ cells. We first assessed stemness using retransfers of T_ML_ cells into infection-time matched secondary recipients. WT and PD-1 KO T_ML_ cells (derived from straight transfers) were isolated at d28 p.i., mixed and adoptively transferred into chronically infected secondary Vβ5 recipients (**Supplementary Figure S3A**). Twenty-eight days later (d28 + 28), the contribution of PD-1 KO cells (94.0% KO) to the P14 compartment was considerably increased compared to the input (51% KO) (**Supplementary Figure S3B**). While the contribution of PD-1 KO cells to the T_ML_ compartment (53% KO) was similar to input (51% KO), the contributions of KO cells to the trans T_EX_ (80.3%) and term T_EX_ compartments (95.8%) were increased (**Supplementary Figures S3C, D**). Although their generation was increased, PD-1 KO T_ML_ cells had a normal self-renewal but an increased capacity to yield more differentiated T_EX_ cells.

**Figure 2:**
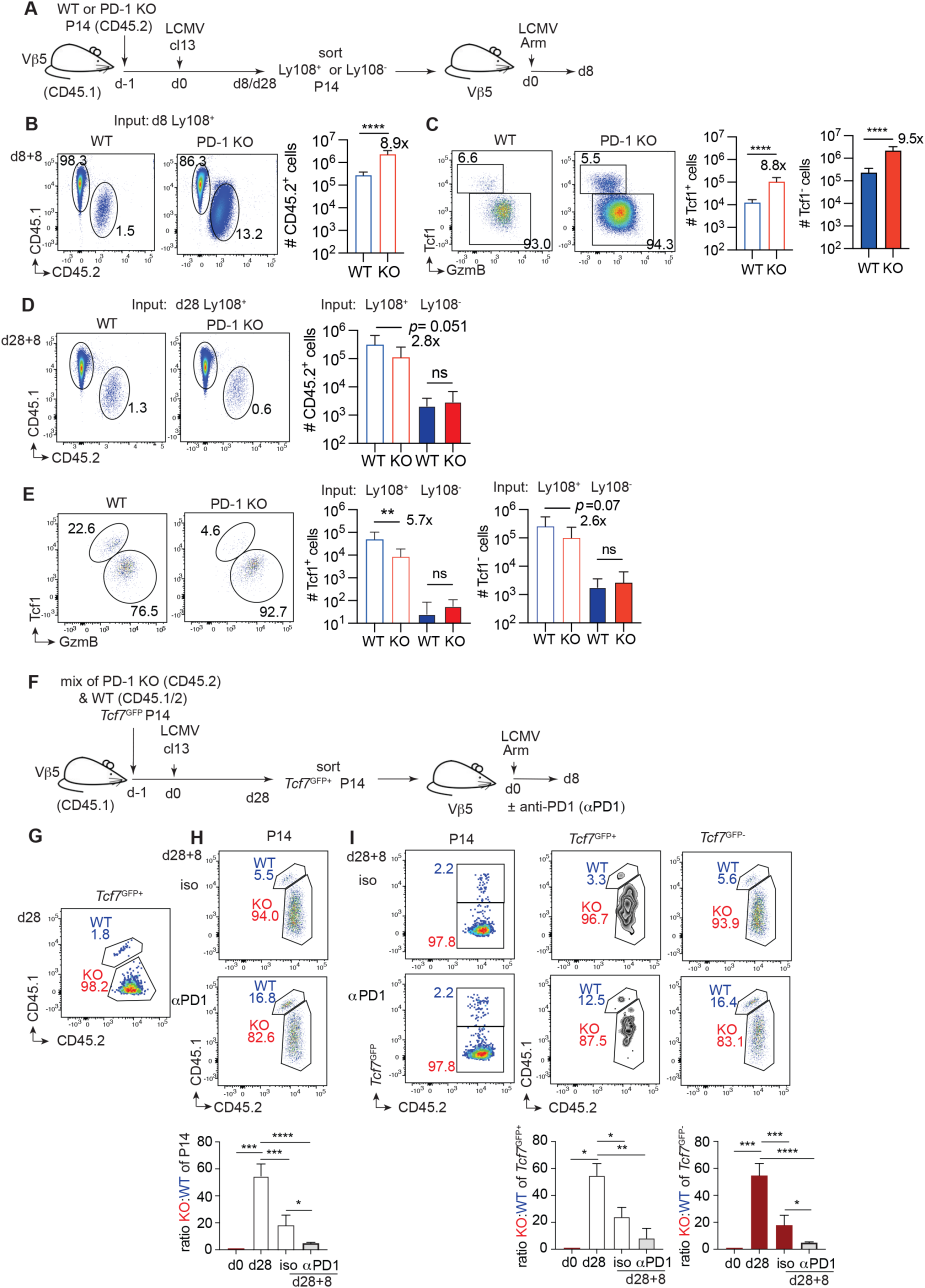
Reduced self-renewal of PD-1 KO T_ML_ cells in response to recall stimulation. **(A–C)** WT or PD-1 KO P14 cells (500) (CD45.2) were injected into V*β*5 mice (CD45.1), which were then infected with LCMV cl13. At d8 post infection (p.i.), Ly108^+^ T_ML_ cells (CD45.2) were flow sorted and equal numbers of cells were transferred into new V*β*5 mice that were infected with LCMV Arm and analysed 8 days later (d8 + 8). **(B)** Gated CD8^+^ T cells were analyzed for the presence of WT and PD-1 KO P14 cells (CD45.2^+^) at d8 + 8 and **(C)** gated P14 cells were analyzed for the presence Tcf1^+^ and Tcf1^-^ GzmB^+^ P14 cells at d8 + 8. **(D, E)** WT P14 (5000 cells) or PD-1 KO P14 cells (CD45.2) (500 cells), were injected into V*β*5 mice (CD45.1), which were then infected with LCMV cl13. At d28 p.i., Ly108^+^ T_ML_ and Ly108^-^ T_EX_ cells (CD45.2) cells were flow sorted and equal numbers of cells were transferred into new V*β*5 mice that were infected with LCMV Arm. Secondary recipients were analysed 8 days later (d28 + 8). **(D)** Gated CD8^+^ T cells were analyzed for the presence of WT and PD-1 KO P14 cells (CD45.2^+^) at d28 + 8 **(E)** Gated P14 cells were analyzed for the expression of Tcf1 versus GzmB d28 + 8. **(F–I)** WT *Tcf7*^GFP^ P14 (CD45.1/2) and PD-1 KO *Tcf7*^GFP^ P14 cells (CD45.2) were mixed in equal proportions and transferred into V*β*5 mice (CD45.1) that were then infected with LCMV cl13. At d28 p.i., the mix of WT and PD-1 KO *Tcf7*^GFP+^ T_ML_ cells was flow sorted and transferred into secondary Vβ5 hosts that were infected with LCMV Arm. Secondary Vβ5 hosts were treated with isotype control (iso) or anti-PD-1 mAb and analyzed 8 days later (d28 + 8). **(G)** Gated T_ML_ cells (*Tcf7*^GFP+^ P14) at d28 p.i. were analysed for the relative presence of WT and PD-1 KO cells. **(H)** Contribution of WT and PD-1 KO cells to the P14 compartment at d8 of the recall response (d28 + 8). **(I)** Gated P14 cells at d28 + 8 were separated into *Tcf7*^GFP+^ and *Tcf7*^GFP-^ cells and analyzed for the presence of WT and PD-1 KO P14 cells. Bar graphs in **(H, I)** show the ratio of PD-1 KO to WT P14 cells at d0, d28 and at d28 + 8 in the presence of isotype (iso) or anti-PD-1 (aPD-1) Ab. Data in **(B–C, G–I)** are compiled from n=2 independent experiments with a total of n=10 mice per group. Data in **(D, E)** are compiled from n=3 independent experiments with a total of n=14 mice per group. All bar graphs show means (± SD). Statistics in **(B–E)** Unpaired two-tailed t-test, in **(H, I)** One-way ANOVA with Tukey’s correction. Significance (*p<0.05, **p<0.01, ***p<0.001, **** p<0.0001, ns=not significant p>0.05).

We next tested the capacity of PD-1 KO T_ML_ cells to respond to an acute challenge during recall stimulation. To this end, T_ML_ cells (CD45.2) from LCMV cl13 infected Vβ5 mice (straight transfers) were flow sorted and equal numbers of cells were transferred into naïve secondary Vβ5 recipients (CD45.1) that were then infected with LCMV Arm, which causes acute resolved infection (**Figure 2A**).

PD-1 KO T_ML_ cells (Ly108^+^) isolated at d8 post LCMV cl13 infection yielded on average 8.9-fold more P14 progeny than WT T_ML_ cells in response to recall stimulation (d8 + 8) (**Figure 2B**). Separation of the progeny based on differential Tcf1 and GzmB expression revealed that the abundance of PD-1 KO Tcf1^+^ GzmB^-^ and Tcf1^-^ GzmB^+^ progeny was increased 8.8-fold and 9.5-fold, respectively (**Figure 2C**). Thus, d8 PD-1 KO T_ML_ cells had an increased recall expansion, self-renewal and differentiation capacity compared to WT T_ML_ cells. In contrast, PD-1 KO T_ML_ cells isolated on d28 p.i. yielded on average 2.8-fold fewer P14 progeny than WT T_ML_ cells (**Figure 2D**) whereby secondary Tcf1^+^ GzmB^-^ cells were reduced 5.7-fold and secondary Tcf1^-^ GzmB^+^ cells were reduced 2.6-fold (**Figure 2E**). PD-1 KO and WT T_EX_ cells (Ly108^-^) essentially failed to expand (**Figures 2D, E**), showing that PD-1 expression does not explain why T_EX_ cells do not expand in response to recall stimulation. Thus, chronic phase PD-1 KO T_ML_ cells had reduced recall expansion and regeneration capacity compared to WT T_ML_ cells.

We sought to confirm the reduced recall response using mixed PD-1 KO and WT T_ML_ cells that derived from the same environment (**Figure 2F**). At d28 p.i., the mix of flow sorted WT and PD-1 KO T_ML_ cells (*Tcf7*^GFP+^) was skewed 50-fold in favor of PD-1 KO T_ML_ cells (1.8% WT) (**Figure 2G**). Following the recall response (d28 + 8), the output skew was reduced to 18-fold in favor of PD-1 KO P14 cells (5.5% WT) (**Figure 2H**). The *Tcf7*^GFP+^ and *Tcf7*^GFP-^ progeny were skewed 22-fold and 15-fold, respectively, in favor of PD-1 KO cells (3.3% WT and 5.6% WT) (**Figure 2I**). As the mix had skewed back 2-3-fold towards WT cells, these data confirmed the reduced recall expansion and self-renewal capacity of chronic phase PD-1 KO T_ML_ cells.

It was possible that PD-1 limited the expansion of WT but not of PD-1 KO T_ML_ cells during recall stimulation and, consequently, that the extent of the skew back towards WT cells was underestimated. When the recall response was performed in the presence of a blocking PD-1 antibody, the skew at d28 + 8 was further reduced to 5-fold in favor of PD-1 KO cells (16.8% WT), compared to the 50-fold skew of the input at d28 (1.8% WT) (**Figure 2H**). The *Tcf7*^GFP+^ and *Tcf7*^GFP-^ progeny were skewed 8-fold and 5-fold, respectively, in favor of PD-1 KO cells (12.5% WT and 16.4% WT) (**Figure 2I**). These data confirmed that chronic phase PD-1 KO T_ML_ cells had reduced recall expansion and regeneration capacity compared to WT T_ML_ cells.

### PD-1 KO T_ML_ cells do not have increased exhaustion features

**Figure 3:**
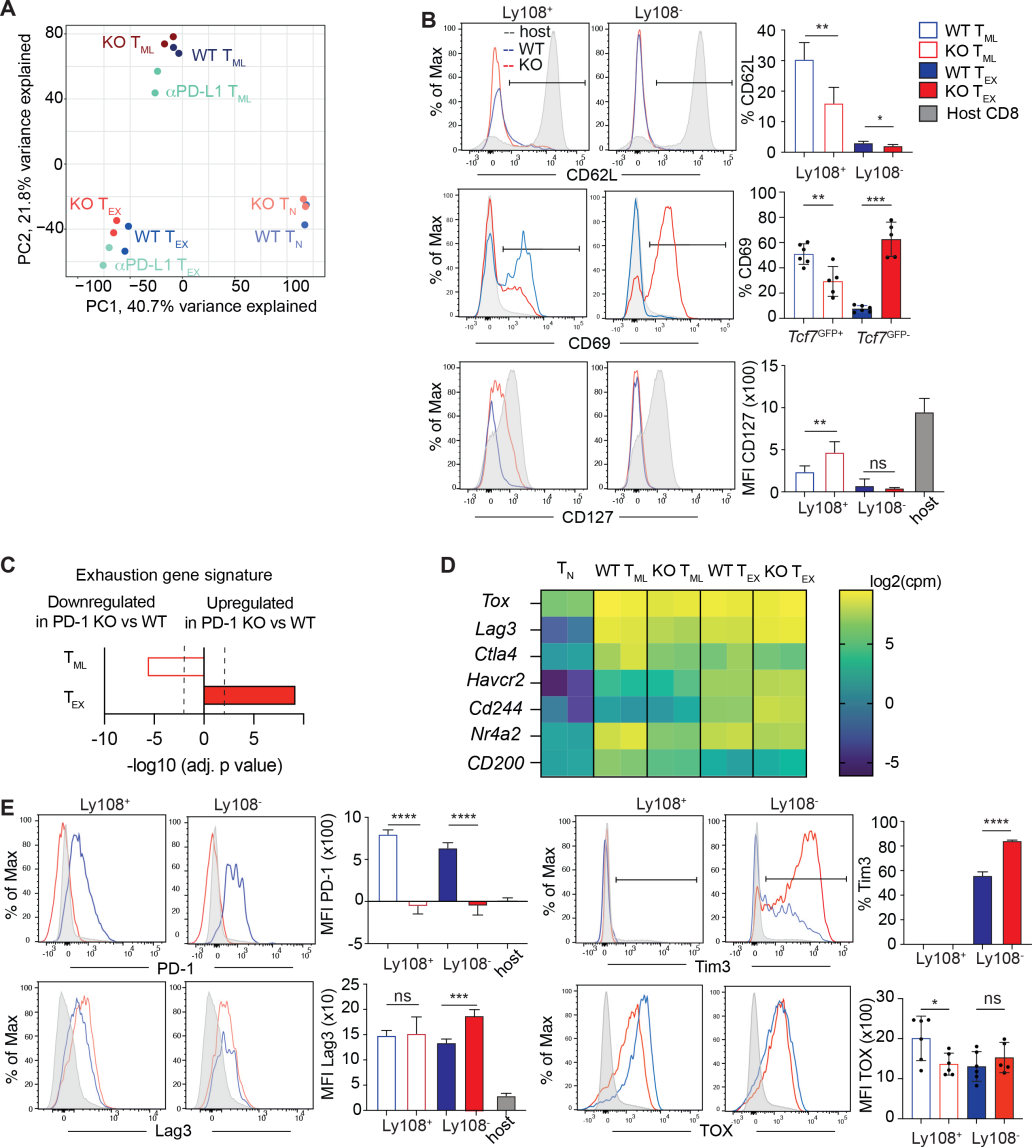
PD-1 KO T_ML_ cells do not increase an exhaustion signature. **(A, C, D)** WT P14 (5000 cells) or PD-1 KO P14 cells (CD45.2) (500 cells) were injected into V*β*5 mice (CD45.1), which were then infected with LCMV cl13. One group of mice receiving WT P14 cells were treated with anti-PD-L1 on d24, d28 and d32 p.i. Ly108^+^ (T_ML_) and Ly108^-^ (T_EX_) P14 cells were flow sorted on d36 p.i. and total cellular RNA was subjected to bulk RNA sequencing. **(A)** Principal Component Analysis (PCA) based on all genes expressed in naïve wild type (WT T_N_) or PD-1 KO P14 cells (KO T_N_), WT T_ML_, KO T_ML,_ T_ML_ cells from anti-PD-L1 treated mice (aPD-L1 T_ML_), WT T_EX_, KO T_EX_ or aPD-L1 T_EX_. Each dot represents a biological replicate. **(B)** Gated P14 cells were divided into T_ML_ (Ly108^+^) and T_EX_ cells (Ly108^-^) and analyzed for the expression of the indicated memory associated receptors relative to CD8^+^ T cells from the Vβ5 host (grey fill). **(C)** Significance of gene set enrichment analysis of PD-1 KO T_ML_ or T_EX_ cells (versus the corresponding WT subset) relative to an exhaustion gene signature (20). The broken line indicates the limit of statistical significance (-log10 (adjusted p-value) =1.3 i.e. p<0.05). **(D)** Heat map indicating the expression (in log_2_(counts per million)) of selected exhaustion associated genes based on the bulk RNAseq analysis. **(E)** Gated P14 cells were divided into T_ML_ (Ly108^+^) and T_EX_ cells (Ly108^-^) and analyzed for the indicated exhaustion markers relative to the CD8^+^ T cells from the Vβ5 host (grey fill). Data in **(A, C, D)** derive from two biological replicates per population. Data in **(B, E)** derive from 4–5 mice per group and are representative of n=2–3 independent experiments. Bar graphs show means (± SD). Statistics: Unpaired two-tailed t-test (between WT versus PD-1KO subsets) whereby *p<0.05, **p<0.01, ***p<0.001, ****p<0.0001, ns, not significant p>0.05).

To begin to address the basis of the reduced self-renewal in response to restimulation and how T_ML_ cells adapted to the lack of PD-1-derived signals, WT and PD-1 KO T_ML_ (Ly108^+^ Tim3^-^) and T_EX_ (Ly108^-^) P14 cells were flow sorted on d28 p.i. and subjected to bulk RNAseq. (The experiment further included P14 subsets from chronically infected mice subjected to PD-L1 blockade (see later)). Principal component analysis (PCA) of the 13’303 retained genes showed that PD-1 KO T_ML_ and WT T_ML_ cells clustered separately from T_EX_ cells (**Figure 3A**). In agreement with these data, PD-1 KO and WT T_ML_ cells differed by n=430 genes while PD-1 KO and WT T_EX_ differed by n=604 genes (**Supplementary Table S1**). Notable genes differentially expressed between PD-1 KO and WT T_ML_ cells included *Sell* (encoding CD62L), *CD69* and *CD127* (*IL7Ra*) (**Supplementary Table S1**). Flow cytometry confirmed that PD-1 KO T_ML_ cells contained smaller subsets of CD62L^+^ and CD69^+^ cells, while the expression level of CD127 was slightly increased (**Figure 3B**). We next addressed whether the PD-1-deficiency was compensated by increases in other exhaustion features. However, if anything, PD-1 KO T_ML_ cells were less enriched in an exhaustion gene signature (20) compared to WT T_ML_ cells (**Figure 3C**). Indeed, several exhaustion-associated genes, including *Tox, Lag3, Ctla4* and *Nr4a2* were expressed at equal or lower levels by PD-1 KO compared to WT T_ML_ cells (**Figure 3D**), which was confirmed for Lag3 and TOX by flow cytometry (**Figure 3E**). In contrast, PD-1 KO T_EX_ cells did have an increased exhaustion signature score (**Figure 3C**), over-expressed certain exhaustion genes (*Lag3*, *Havcr2* (TIM3), *Cd244*) and expressed more Lag3 and TIM3 compared to WT T_EX_ cells (**Figure 3E**, **Supplementary Figure S4A**). Further separation of T_EX_ cells into trans (Ly108^-^ Cx3cr1^+^) and term T_EX_ cells (Ly108^-^ CX3CR1^-^) showed that PD-1 KO T_EX_ cells contained an expanded term and a reduced trans T_EX_ compartment (**Supplementary Figures S4B, C**). As term T_EX_ cells display increased exhaustion features compared to trans T_EX_ cells (19), we quantified the expression of exhaustion markers in the two T_EX_ subpopulations. Indeed, WT and PD-1 KO trans or term T_EX_ cells did not differ in their expression of Lag3 or TOX (**Supplementary Figure S4D**). The increased exhaustion attributes of PD-1 KO cells noted before (16) are thus explained at least in part by a distinct population composition. We conclude that T_ML_ cells did not compensate PD-1-deficiency via the upregulation of other exhaustion-associated molecules.

### PD-1 KO T_ML_ cells have reduced constitutive expression of NFkB and NFAT gene signatures

**Figure 4:**
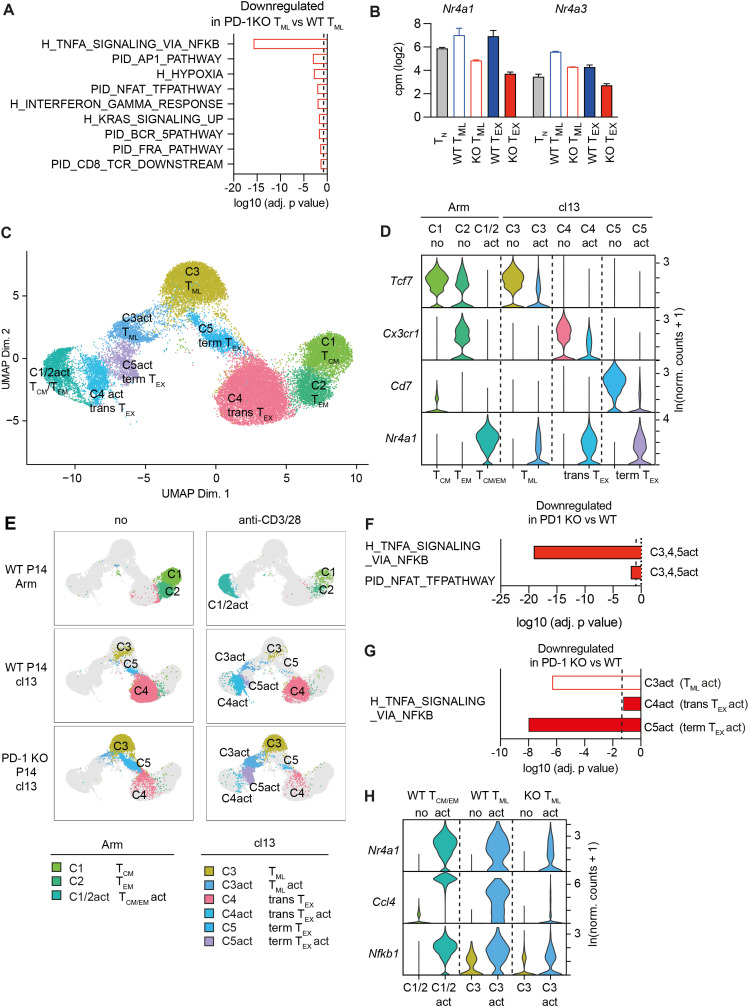
Reduced TCR-mediated induction of NFAT and NFkB gene signatures in PD-1 KO T_ML_ cells. **(A)** Genes differentially expressed between PD-1 KO versus WT T_ML_ cells were subjected to gene set enrichment analysis (GSEA) using the Pathway Interaction Database (PID) and Hallmark collections. The bar graph depicts the significance of pathways downregulated in PD-1 KO vs WT T_ML_ cells (-log10 adjusted p-value). **(B)** Expression of *Nr4a1* (Nur77) and *Nr4a3* based on the bulk RNAseq analysis. **(C–H)** WT and PD-1 KO P14 cells (d28 post LCMV cl13 infection) or conventional memory P14 cells (d28 post LCMV Arm infection) were flow sorted and left unstimulated (“no”) or restimulated with anti-CD3/28 *in vitro* for 4 h and then subjected to scRNAseq analysis. **(C)** UMAP projections of rested and activated cells colored according to their cluster annotation, as assigned based on the expression of key markers genes shown in **(D)**: Conventional memory cells: C1 T_CM_, C2 T_EM_. Chronic infection: C3 T_ML_, C4 transitory T_EX_ (trans T_EX_), C5 terminal T_EX_ (term T_EX_). The corresponding populations that showed signs of activation based on *Nr4a1* upregulation are referred to as “act”. **(E)** UMAP projection split per individual library and including cluster annotations. **(F)** Genes differentially expressed by total activated PD-1 KO versus WT P14 cells were subjected to overrepresentation analysis (ORA) and analyzed for the enrichment of the TNF_NFkB and NFAT gene signatures **(G)** Genes differentially expressed by activated PD-1 KO T_ML_ cells (C3act), trans T_EX_ (C4act) and term T_EX_ (C5act) cells relative to the corresponding activated (act) WT subsets were subjected to GSEA and analyzed for the enrichment of the TNF_NFkB gene signature. The broken line in **(F, G)** indicates the limit of statistical significance (-log10(adjusted p-value)) = 1.3 i.e. p<0.05). **(H)** The violin plot shows the expression of selected TNF_NFkB signature genes in the indicated populations of unstimulated (no) and anti-CD3/28 activated cells (act). Data in **(A, B)** derive from two biological replicates per population. Data in **(C–H)** derive from a single experiment, whereby the number of cells analyzed per library and cluster is indicated in **Supplementary Table S3**.

To identify transcriptional changes indicative of a functional adaptation of PD-1 KO T_ML_ cells, we subjected differentially expressed genes to gene set enrichment analyses (GSEA) against the Pathway Interaction Database (PID) and the Hallmark gene signatures (H) (MsigDB). While no gene signatures were upregulated, the Hallmark TNFA_via_NFkB (referred to as NFkB signature hereafter) pathway was the most downregulated signature in PD-1 KO relative to WT T_ML_ cells (**Figure 4A**). The top 4 downregulated pathways further included the “PID_AP1” and “PID_NFAT” signatures (**Figure 4A**) (**Supplementary Table S2**), key signaling pathways downstream of the TCR, indicating globally reduced TCR signaling in PD-1 KO T_ML_ cells. Corresponding data were obtained for T_EX_ cells (**Supplementary Figure S5A**) (**Supplementary Table S2**). In agreement with reduced TCR signaling, PD-1KO T_ML_ cells had a reduced overlap with the PID_CD8_TCR downstream pathway (**Figure 4A**) and *Nr4a1* (Nur77) and *Nr4a3*, which report the strength and the duration of TCR signaling (21, 22) were both reduced in PD-1 KO compared to WT T_ML_ as well as in T_EX_ cells (**Figure 4B**). These data suggested that T_ML_ cells, which cannot express PD-1, have globally reduced TCR downstream signaling independent of changes in exhaustion.

### Reduced TCR-mediated induction of NFkB and NFAT gene signatures in PD-1 KO T_ML_ cells

To confirm and extend the above findings we next addressed the transcriptional response of PD-1 KO cells to acute TCR restimulation. WT and PD-1 KO P14 cells were isolated from chronically infected mice (WT cl13 and KO cl13) or from LCMV immune mice (WT Arm). The cells were either rested or reactivated with antibodies to CD3 and CD28 (aCD3/28) *in vitro* (in the absence of other cells or PD-1 engagement) for 4h before analysis by single cell RNA sequencing (scRNAseq). Unsupervised clustering following uniform manifold approximation and projection (UMAP) representation identified 11 discrete clusters of cells. A cluster with <500 cells (C11) and cycling cells (*mKi67^+^*) (C10) were excluded from further analysis (**Figure 4C**, **Supplementary Figure S5B**). In rested P14 cells from chronically infected mice, differential expression of key marker genes (*Tcf7, Cx3cr1* and *Cd7*) allowed the identification of T_ML_ (C3), transitory T_EX_ (trans T_EX_) (C4) and terminal T_EX_ cells (term T_EX_) (C5). T_CM_ (C1) and T_EM_ cells (C2) were identified among P14 cells from LCMV Arm immune mice (**Figures 4C, E**) (**Supplementary Table S3**). In response to CD3/28 restimulation, all samples yielded additional cell clusters, which corresponded to activated versions of the above CD8^+^ T cell subsets, as judged by the expression of the above markers combined with the upregulation of *Nr4a1* (**Figures 4C-E**, **Supplementary Figure S5C**) (**Supplementary Table S3**). While most LCMV Arm memory cells (T_CM_/T_EM_) showed signs of activation (86.4%), fewer WT T_ML_ (66.1%) and even fewer PD-1 KO T_ML_ cells (52.9%) had responded to stimulation (**Supplementary Table S3**).

Pathway analysis showed that CD3/28 stimulation induced the NFkB, NFAT and AP-1 gene signatures in T_CM_/T_EM_ cells (C1/2act vs C1/2) (**Supplementary Figure S5D**) (see **Supplementary Table S4** for differentially expressed genes (DEG) and **Supplementary Table S5** for the result of the pathway analysis), demonstrating that these gene signatures are indeed induced upon TCR engagement. We next compared the TCR-induced transcriptional response of P14 cells derived from chronic infection (combined C3,4,5act). This showed a considerably weaker induction of the NFkB and a modestly weaker induction of the NFAT signature in PD-1 KO compared to WT P14 cells (**Figure 4F**) (**Supplementary Table S4**, **Supplementary Figure S5**). The AP-1 signature was not different. As the cellular composition of the PD-1 KO and WT cells differed, we next compared the transcriptional response of the corresponding subsets. This confirmed a weaker induction of the NFkB signature in PD-1 KO T_ML_ (C3act), transitory T_EX_ cells (C4act) and terminal T_EX_ cell (C5act) compared to the corresponding WT subsets (**Figure 4G**) (**Supplementary Table S5**). The smaller difference in transitory T_EX_ cells is likely due to the low number of PD-1 KO cells. Differentially induced NFkB signature genes in T_ML_ cells included *Nr4a1, Ccl4 and Nfkb1* (**Figure 4H**). The induction of the NFAT and AP-1 signatures did not differ between PD-1 KO and WT subsets, which was likely related to the smaller initial difference and the low number of cells in some of the populations. The data thus showed that PD-1 KO T_ML_ cells suffered from a reduced TCR-mediated induction of an NFkB gene signature. The changes induced in response to acute TCR engagement thus corresponded to those of the constitutive gene expression patterns, suggesting a reduced ability of the TCR to activate PD-1 KO T_ML_ cells, accounting for the reduced recall expansion.

### Impaired TCR-mediated activation of NFkB, NFAT and Ca^2+^ flux in PD-1 KO T_ML_ cells

**Figure 5:**
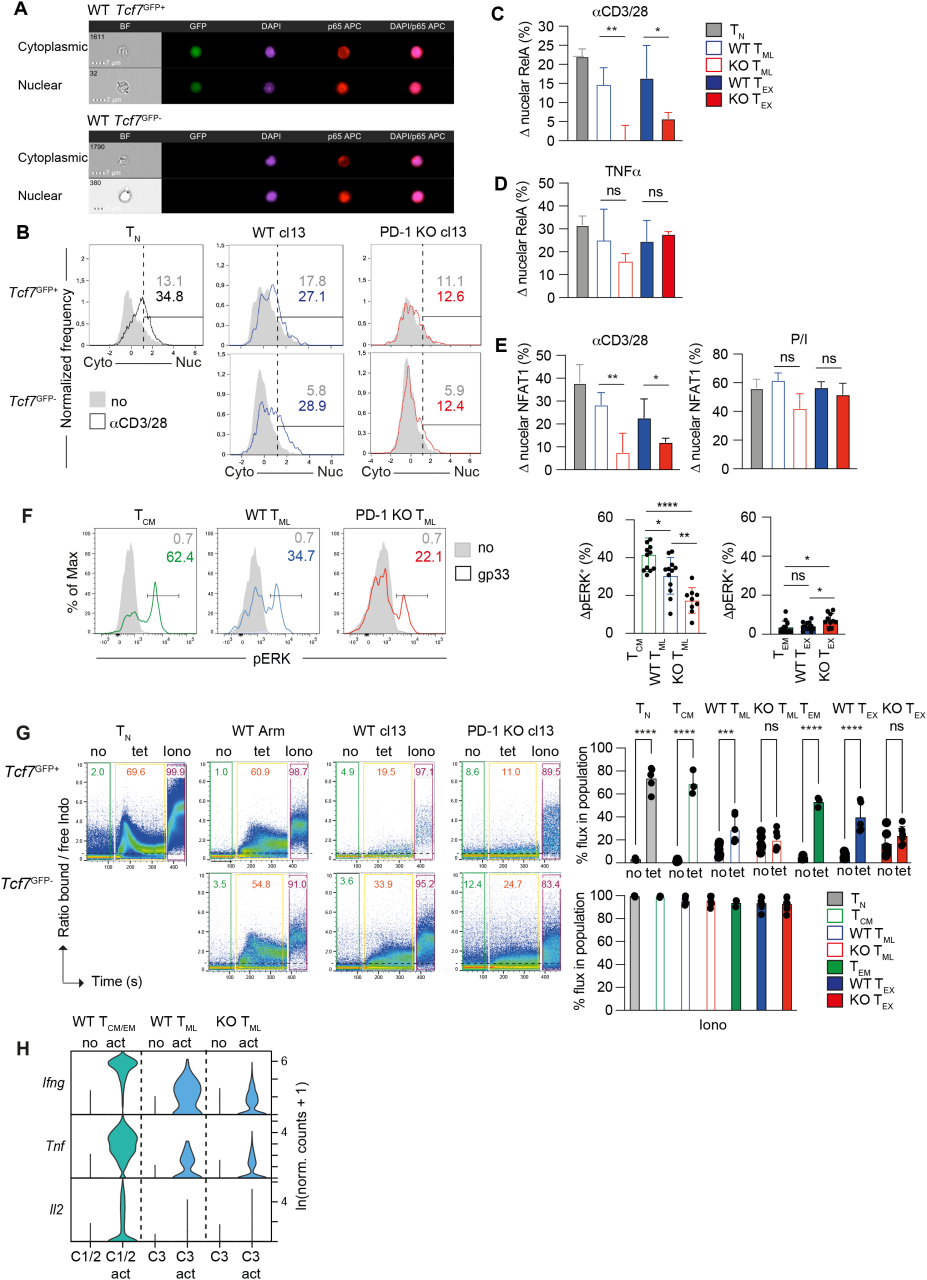
Reduced activation of NFkB, NFAT and ERK in PD-1 KO T_ML_ cells. **(A–E)** WT or PD-1 KO *Tcf7*^GFP^ P14 cells at d28 post LCMV cl13 infection or naïve WT P14 cells (T_N_) were flow sorted and rested (no) or restimulated *in vitro* with anti-CD3/28 antibodies for 4 h and then stained with RelA (p65) or NFAT1 Abs and the nuclear dye DAPI. **(A)** Imagestream analysis, showing examples of cytoplasmic (top) or nuclear (bottom) localization of RelA (bottom) in gated *Tcf7*^GFP+^ (T_ML_) and *Tcf7*^GFP-^ (T_EX_) P14 cells. Original magnification 340. **(B)** T_ML_ and T_EX_ cells were analyzed for the co-localization (similarity) of DAPI and RelA staining. Overlays show the similarity in rested (grey fill) compared to anti-CD3/28 stimulated cells (open histograms). **(C, D)** The bar graphs show the percent of cells with nuclear RelA (similarity >1) in response to **(C)** anti-CD3/28 or **(D)** TNFa stimulation in T_ML_ and T_EX_ cells whereby the values from the corresponding rested cells were subtracted (termed: Δ%). **(E)** The bar graphs show the percent of cells with nuclear NFAT1 (similarity >1) in response to anti-CD3/28 or P/I in T_ML_ and T_EX_ cells. The values from the corresponding rested cells were subtracted (Δ%). **(F, G)** CD8^+^ T cells containing WT or PD-1 KO *Tcf7*^GFP^ P14 cells were purified from the spleen of LCMV cl13 or LCMV Arm infected mice at d28 p.i. **(F)** Cells were rested (no) or stimulated using gp33 peptide for 2 to 4h. Gated *Tcf7*^GFP+^ and *Tcf7*^GFP-^ P14 cells (CD45.2^+^) were analyzed for phospho ERK (pERK). The bar graphs show the % of pERK^+^ cells, whereby values form rested cells were subtracted (Δ%). **(G)** Cells were loaded with Indo1 and subjected to Ca^2+^ flux analysis. The dot plots show the ratio of free to bound Indo1 in gated *Tcf7*^GFP+^ or *Tcf7*^GFP-^ P14 cells (CD45.2^+^) in the absence of stimulation (no), following the addition of gp33-tetramer (tet) or the addition of Ionomycin (Iono) over time. The bar graphs show the % cells with an increased ratio of bound/free Indo1. **(H)** The violin plot shows the expression IFNγ, TNFα and IL2 transcript levels in the indicated populations of unstimulated (no) and anti-CD3/28 activated cells (act) based on scRNAseq. Data in **(A–E, F)** are compiled from 2–4 independent experiments with a total of n=3–6 determinations. Data in **(G)** are single determinations compiled from 3–6 independent experiments. Data in **(H)** derive from a single experiment, whereby the number of cells analyzed per library and cluster is indicated in **Supplementary Table S3**. All bar graphs show means (± SD). Statistics in **(A–E, G)** Unpaired two-tailed t-test to determine significance between the corresponding PD-1 KO and WT populations, in **(F)** One-way ANOVA. Significance (*p<0.05, **p<0.01, ***p<0.001, ****p<0.0001, ns, not significant p>0.05).

To confirm the reduced induction of a NFkB gene signature and to address its basis, we tested the capacity of CD3/28 stimulation to induce nuclear translocation of RelA (p65), a key transcription factor of the canonical NFkB pathway in WT and PD-1 KO T_ML_ and T_EX_ cells. Nuclear localization was defined by a positive similarity score, representing the relative colocalization of the nuclear dye DAPI and RelA (p65) in imaging flow cytometry. RelA (p65) translocation in response to CD3/28 engagement was readily observed in WT T_ML_ and T_EX_ cells but did not occur in the corresponding PD-1 KO subsets (**Figures 5A–C**). This was not due to a difference in total RelA levels (**Supplementary Figure S6A**). Stimulation with the proinflammatory cytokine TNFα, which bypasses TCR proximal signaling, induced comparable RelA translocation in WT and PD-1 KO cells (**Figure 5D****).** We similarly used imaging flow cytometry to estimate NFAT activation. While CD3/28 stimulation mediated nuclear translocation of NFAT1 in WT T_ML_ and T_EX_ cells, this was significantly reduced in the corresponding PD-1 KO subsets. NFAT1 expression and NFAT1 nuclear translocation in response to P/I were comparable (**Figure 5E**, **Supplementary Figure S6B**). Finally, we estimated the activation of ERK, which is critical for the activation of AP-1. In response to TCR stimulation, the fraction of cells with phosphorylated ERK (pERK^+^) was significantly reduced among PD-1 KO compared WT T_ML_ cells (**Figure 5F**). ERK was not activated in T_EX_ cells (**Figure 5F**). The data suggested that PD-1 KO T_ML_ cells suffered from a reduced activation of the NFkB, NFAT and AP-1 pathways in response to TCR/CD28 stimulation.

To further define the defect in PD-1 KO cells, we investigated intracellular Ca^2+^ mobilization, an essential signal for both NFAT and NFkB activation (23). Compared to naïve P14 cells (T_N_) or T_CM_ cells, gp33 tetramer-induced Ca^2+^ flux was reduced in WT T_ML_ cells and completely blunted in PD-1 KO T_ML_ cells (**Figure 5G**). PD-1 KO T_ML_ cells showed an increased basal level of cytoplasmic free Ca^2+^, but no further Ca^2+^ mobilization in response to tetramer stimulation. Corresponding data were obtained in T_EX_ cells. Ionomycin-induced Ca^2+^ flux was comparable among the different sub populations (**Figure 5G**). The reduced activation of PD-1 KO T_ML_ and T_EX_ cells in response to TCR triggering was not based on a difference in TCR expression levels (**Supplementary Figure S6C**) or TCR ligand binding capacity, as judged by comparable gp33 tetramer binding (**Supplementary Figure S6D**). Thus, deficient Ca^2+^ mobilization in PD-1 KO cells was due to a defect in TCR signaling.

A key consequence of TCR stimulation and the cooperation of the NFkB, NFAT and AP-1 pathways is the production of cytokine mRNAs. Restimulation with antiCD3/28 Abs induced considerably less IFNγ and TNFα mRNA in PD-1 KO T_ML_ compared to WT T_ML_ cells (**Figure 5H**). Despite the reduced transcript levels IFNγ and TNFα protein production was comparable (**Supplementary Figures S6E, F**), raising the possibility that PD-1 impacts cytokine mRNA translation.

While gp33-driven cytokine production by PD-1 KO and WT T_ML_ cells was lower compared to T_CM_ cells, it considerably improved in response to P/I stimulation (**Supplementary Figure S6F**). Thus, the limited cytokine production by PD-1 KO (and WT) T_ML_ cells was related to TCR signaling defect at or upstream of PKC activation and Ca^2+^ mobilization, consistent with negative effects on all major downstream pathways.

### The reduced recall response of PD-1 KO T_ML_ cells correlates with reduced *Klf4* and *Myb* expression.

**Figure 6:**
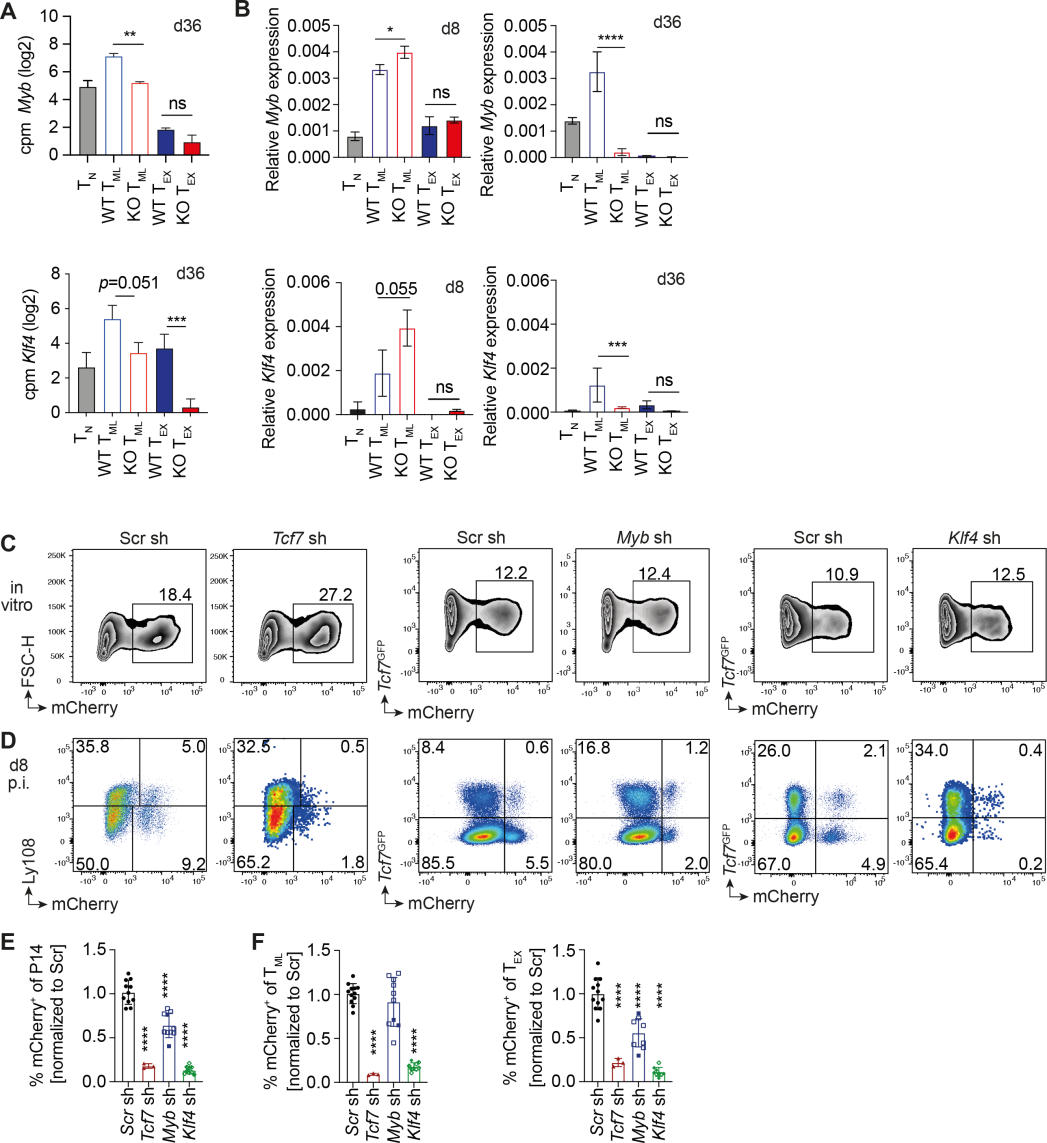
Basis for the reduced stemness of PD-1 KO T_ML_ cells. **(A)** Expression of *Myb* and *Klf4* in WT and PD-1 KO T_ML_ and T_EX_ cells at d36 p.i. based on bulk RNAseq analysis. **(B)** Expression of *Myb* and *Klf4* in WT and PD-1 KO T_ML_ and T_EX_ cells on d8 and d36 p.i. based on RT-qPCR analysis. **(C–F)** P14 or *Tcf7^DTR-^*^GFP^ P14 cells (referred to as *Tcf7*^GFP^) were transduced with Lentiviral vectors encoding gene-specific short hairpin (sh) RNA constructs or a scrambled (scr) control sh RNA. **(C)** Expression of *Tcf7* sh, *Myb* sh or *Klf4* sh constructs (mCherry^+^) versus forward scatter (FSC) or *Tcf7*^GFP^*in vitro.***(D)** Presence of sh-expressing (mCherry^+^) Ly108^+^ or *Tcf7*^GFP+^ cells among gated P14 cells at d8 post LCMV cl13 infection. **(E)** Fraction of mCherry^+^ cells among P14 cells (normalized to the scr control). **(F)** Fraction of mCherry^+^ cells among T_ML_ cells (left) or among T_EX_ cells (right) (both normalized to the scr control). Data in **(A, B)** derive from a single experiment with two biological replicates per population. Data in **(C–F)** are compiled from 2–3 independent experiments with a total of 3 or more determinations for each sh construct. All bar graphs show means (± SD). Statistics in **(A, B)** Unpaired two-tailed t-test to determine significance between the corresponding PD-1 KO and WT populations, in **(C–F)** One-way ANOVA comparing the mean of each column to scr sh using uncorrected Fisher’s LSD test. Significance (*p<0.05, **p<0.01, ***p<0.001, **** p<0.0001, ns=not significant p>0.05).

We next used the TCR downstream, AP-1, NFAT and Nfkb gene signatures to search for transcription factors that are involved in the formation and/or function of T_ML_ cells and whose expression was altered in PD-1 KO T_ML_ cells. We found reduced expression of the PID_AP-1 signature gene *Myb* and the Nfkb signature gene *Klf4* in PD-1 KO compared to WT T_ML_ cells (**Figure 6A**, **Supplementary Figures S7A, B**). *Myb*-deficiency reduces the formation and function of conventional memory CD8^+^ T cells arising in response to acute resolved infection (24) and precludes the formation of T_ML_ cells in chronic infection (25). Klf4 ensures the stemness of central memory precursor cells, which arise in response to acute resolved infection (26). Stimulation of T_N_ cells via the TCR *in vitro* increased *Myb* and transiently downregulated *Klf4* expression (not depicted) in agreement with (25, 27).

Relative to T_N_ cells, *Klf4* and *Myb* were upregulated in both WT and PD-1 KO T_ML_ cells at the acute phase of chronic infection (d8 p.i) based on RT-qPCR analysis (**Figure 6B**). *Klf4* and *Myb* levels were maintained in chronic phase WT T_ML_ cells, but had declined in PD-1 KO T_ML_ cells at d28 (**Figure 6B**), correlating with the reduced TCR signal transduction. Reduced *Myb* expression in PD-1 KO T_ML_ cells correlated with a reduced fraction of CD62L^+^ T_ML_ cells (**Figure 3B**), consistent with an absence of self-renewing CD62L^+^ T_ML_ cells in *Myb*-deficient mice (25).

We next addressed whether reduced *Myb* or *Klf4* expression accounted for the reduced stemness of PD-1 KO T_ML_ cells. To this end we performed *Myb* and *Klf4* knock-down experiments in WT cells using lentiviral (LV) sort hairpin (sh) RNA constructs. We opted for a knock down rather than a knock out approach in order to reproduce the partial reduction of gene expression seen in the PD-1 KO cells. *In vitro* experiments showed that shRNA constructs reduced *Myb* and *Klf4* expression by at least 50% compared to control scr sh transduced cells (**Supplementary Figure S7C**) (26).

In any given experiment, the different LV preparations yielded comparable fractions of transduced (mCherry^+^) WT or *Tcf7*^GFP^ P14 cells *in vitro* (**Figure 6C**). LV-transduced P14 cells were then transferred into Vβ5 mice that had been infected with LCMV cl13 one day earlier. To validate the approach, we addressed whether *Tcf7* knock down recapitulated the deficient generation of T_ML_ cells previously observed using *Tcf7*^-/-^ P14 cells (17). At d8 p.i., the fraction P14 cells expressing a *Tcf7* shRNA construct was reduced about 6-fold compared to a scrambled (scr) Sh control construct (**Figures 6D, E**). The reduction was evident in both T_ML_ as well as T_EX_ cells (**Figure 6F**). This validated our approach to functionally test stemness genes in chronic viral infection.

Transduction with *Myb* shRNA did not alter the presence of T_ML_ cells but reduced their differentiation into T_EX_ cells at d8 p.i. (**Figures 6D–F**). Thus, reduced *Myb* expression impaired the differentiation of T_ML_ cells. In contrast, the expression of *Klf4* shRNA almost completely prevented the generation of T_ML_ cells and T_EX_ cells at d8 p.i (**Figures 6D–F**). Thus, partially reduced *Myb* and *Klf4* affected the differentiation and the generation of T_ML_ cells, in line with the reduced stemness of PD-1 KO T_ML_ cells in response to recall stimulation.

### PD-L1 blockade reduces the self-renewal capacity of T_ML_ cells

**Figure 7:**
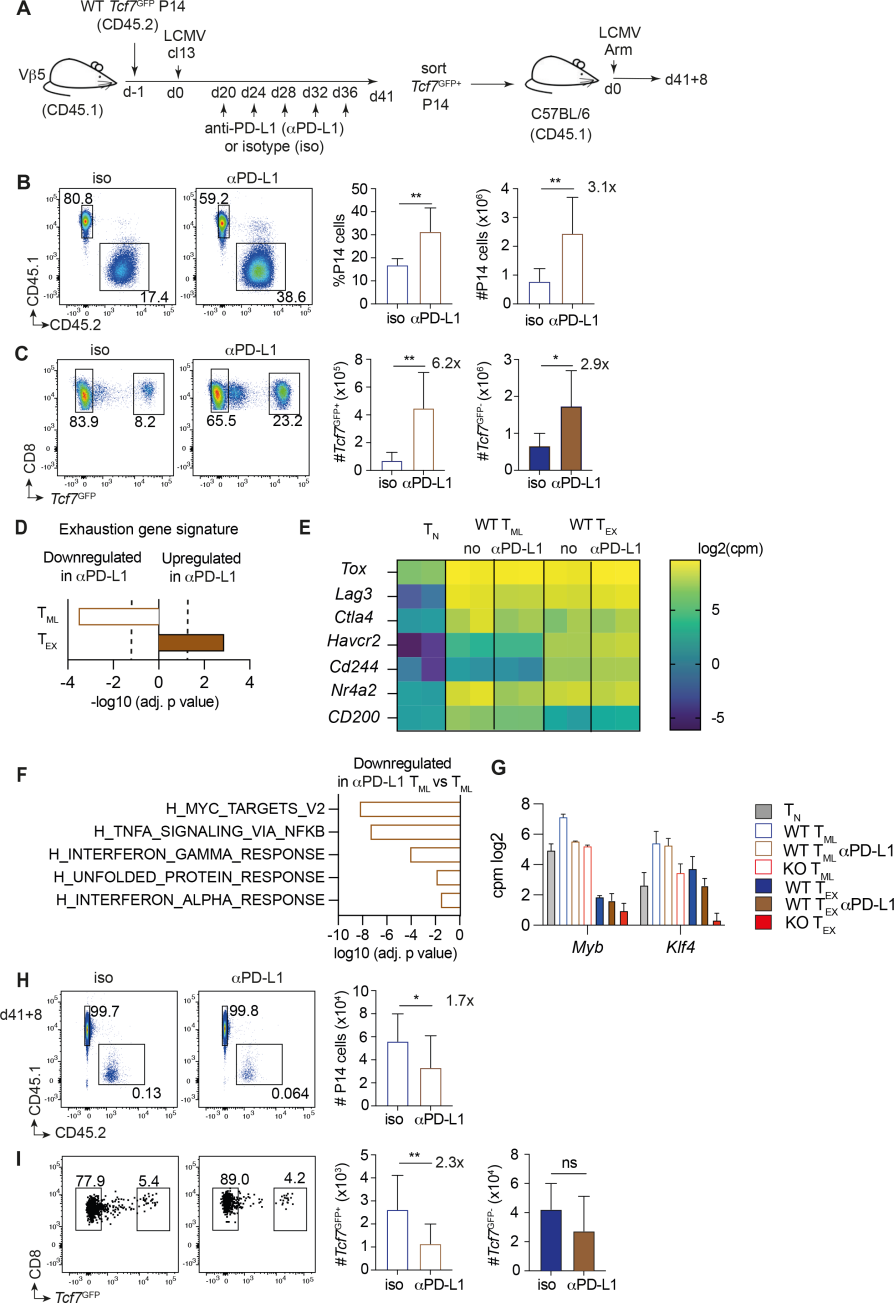
Transient PD-L1 blockade reduces the self-renewal capacity of T_ML_ cells. **(A–C)** Naïve *Tcf7*^GFP^ P14 cells (CD45.2) were injected into V*β*5 mice (CD45.1), which were then infected with LCMV cl13. Starting at d20 p.i., mice were treated with anti-PD-L1 (aPD-L1) or isotype control mAb, 5 times every 4^th^ day. Mice were analyzed 5 days after the last injection on d41 p.i. **(B)** Presence of P14 cells (CD45.2^+^) among gated CD8^+^ T cells in the spleen of aPD-L1 or isotype (iso) treated mice at d41 p.i. **(C)** Gated P14 cells (CD45.2^+^) were analyzed for the presence of *Tcf7*^GFP+^ cells. **(D–G)** Bulk RNAseq analysis of anti-PD-L1 and control WT T_ML_ and T_EX_ cells. **(D)** Significance of gene set enrichment analysis of anti-PD-L1 treated T_ML_ or T_EX_ cells (versus the corresponding non-treated subset) relative to an exhaustion gene signature (20). The broken line indicates the limit of statistical significance (-log_10_(adjusted p-value) = 1.3 i.e. p<0.05). **(E)** Expression of selected exhaustion-associated genes, in log_2_(counts per million), based on bulk RNAseq. The untreated controls correspond to the samples shown in **Figure 3A**. **(F)** Genes differentially expressed between anti-PD-L1 treated and untreated T_ML_ cells were subjected to gene set enrichment analysis (GSEA) using the PID and Hallmark collections. The bar graph shows the top pathways downregulated in aPD-L1 treated T_ML_ cells (log_10_(adjusted p-value)). **(G)** Expression of *Myb* and *Klf4* in the indicated subsets based on bulk RNAseq. **(A, H, I)** At d41 p.i. *Tcf7*^GFP+^ P14 cells (CD45.2) from anti-PD-L1 treated and isotype control treated Vβ5 hosts were flow sorted and transferred into new B6 hosts (CD45.1) that were infected with LCMV Arm. Secondary recipients were analysed 8 days later (d41 + 8). **(H)** Presence of P14 cells (CD45.2^+^) among gated CD8^+^ T cells in the spleen at d41 + 8. **(I)** Gated P14 cells (CD45.2^+^) were analyzed for the presence of *Tcf7*^GFP+^ cells. Data in **(B, C)** derive from n=4–8 mice per group and are representative of n=3 independent experiments. Data in **(D–G)** derive from 2 biological replicates. Data in **(H, I)** are compiled from n=2 independent experiments with a total of n=10–15 mice. All bar graphs show means (± SD). Statistics in **(B, C, H, I)** Unpaired two-tailed t-test. Significance (*p<0.05, **p<0.01, ns=not significant p>0.05).

As the continuous absence of PD-1 altered the stemness of T_ML_ cells, we next addressed whether transient blockade of the PD-1/PD-L1 interaction had similar effects on T_ML_ cells. We transferred *Tcf7*^GFP^ P14 cells (CD45.2) into Vβ5 mice (CD45.1) prior to infection with LCMV cl13. Twenty days later we started to treat recipient mice with anti-PD-L1 or isotype control Ab. Recipients were analyzed 5 days after the last injection i.e. on d41 p.i. (**Figure 7A**). Spleens of recipients treated with anti-PD-L1 contained 3.1-fold more P14 cells than control animals (**Figure 7B**). The abundance of T_ML_ cells (*Tcf7*^GFP+^) was increased 6.2-fold (**Figure 7C**) in agreement with (28). At this stage, IFNγ and TNFα production was no longer elevated (**Supplementary Figure S8A**), as seen when tested at earlier time points after the last dose (3). Thus, the re-activation in response to checkpoint blockade had mostly ceased 5 days after the last anti-PD-L1 dose.

We next compared the transcriptomes of WT T_ML_ (Ly108^+^ Tim3^-^) and T_EX_ (Ly108^+^ Tim3^-^) cells that had been subjected to PD-L1 blockade (**Figure 3A**). Anti-PD-L1-treated and control WT T_ML_ cells differed by more genes (n=759) than PD-1 KO and WT T_ML_ cells (n=431 genes) (adjusted p value <0.05) (**Supplementary Table S1**). A total of 160 differentially expressed genes were shared by PD-1 KO and anti-PD-L1-treated WT T_ML_ cells (**Supplementary Table S1**). Anti-PD-L1 treated T_EX_ cells were even more distinct (n=1021 DEG) (**Supplementary Table S1**).

Similar to PD-1 deficiency, PD-L1 blockade reduced rather than increased the expression of an exhaustion gene signature of WT T_ML_ cells (**Figure 7D**). Indeed, several exhaustion-associated genes, including *Tox, Lag3, Ctla4* and *Nr4a2* were expressed at equal or lower levels by anti-PD-L1 treated compared to untreated T_ML_ cells (**Figure 7E**), which was confirmed by flow cytometry for Lag3 (**Supplementary Figure S8B**). On the other hand, PD-1 expression was increased on WT T_ML_ cells (**Supplementary Figure S8B**), as expected when ligand engagement is prevented. Thus, PD-L1 blockade did not result increase exhaustion features in T_ML_ cells. Conversely, T_EX_ cells from PD-L1 treated mice showed an increased exhaustion score. Indeed, Lag3 and Tim3 expression were increased (**Supplementary Figure S8B**). The increased exhaustion in T_EX_ cells was explained in part by a reduced contribution of transitory versus terminal T_EX_ cells to the total T_EX_ compartment (**Supplementary Figure S8C**). On the other hand, PD-L1 blockade resulted in a reduction of the NFkB gene signature in T_ML_ cells (**Figure 7F**) (**Supplementary Table S2**), similar to PD-1 KO T_ML_ cells. T_ML_ cells from anti-PD-L1-treated mice also expressed less *Myb*, while *Klf4* expression was unchanged (**Figure 7G**).

Finally, we addressed the capacity of T_ML_ cells derived from anti-PD-L1-treated mice to mount a recall response. The recall expansion of T_ML_ cells derived from anti-PD-L1-treated mice was reduced 1.7-fold compared to that from untreated controls (**Figure 7H**), whereby the generation of secondary of *Tcf7*^GFP+^ cells was reduced 2.3-fold (**Figure 7I**). Thus, the transient interruption of PD-1 signaling during the chronic phase of the infection does have a negative effect on the stemness of T_ML_ cells.

## Discussion

Here we have addressed whether and how T_ML_ cells, which have stem-like properties, adapt to chronic stimulation if they cannot express PD-1. We found that PD-1 KO T_ML_ cells had reduced capacity to regenerate in response to acute recall stimulation. The absence of PD-1 was not compensated by an increased expression of other inhibitory receptors or exhaustion related genes. Rather, the TCR of PD-1 KO T_ML_ cells had a reduced capacity to activate these cells. Thus, PD-1 was needed to preserve the ability of T_ML_ cells to regenerate in response to rechallenge.

In the course of chronic viral infection, PD-1-deficient T_ML_ cells were generated considerably more efficiently than WT and then persisted surprisingly stably for extended periods of time. PD-1-deficient T_EX_ cells were also generated more efficiently, but these cells started to decline at >d200 of the infection, in line with the reduced presence of PD-1 KO P14 cells in CD4-depleted hosts at >d350 of the infection (16). Despite the increased early generation (e.g. on d28), these PD-1 KO T_ML_ cells did not persist more efficiently than WT T_ML_ cells, reinforcing the notion that PD-1 KO T_ML_ cells efficiently regenerate during chronic infection. However, when chronic phase PD-1 KO T_ML_ cells (d28 p.i) were acutely restimulated (during a recall response), their expansion/regeneration was reduced compared to WT. That contrasted with the increased expansion/regeneration of acute phase PD-1 KO T_ML_ cells (d8 p.i). Thus, the expansion/regeneration capacity of PD-1 KO T_ML_ cells in response to acute restimulation erodes over time. In contrast, the expansion/differentiation capacity of PD-1 KO T_ML_ cells in response to acute restimulation (as a function of their abundance) corresponded to WT at the early phase, but was actually increased at the intermediate phase and may decline at the late stage.

We next addressed the basis for the reduced regeneration of PD-1 KO T_ML_ cells in response to acute restimulation. We first considered the possibility that T_ML_ cells compensate the absence of PD-1 by an increased expression of other inhibitory receptors or other exhaustion-related molecules. However, we did not obtain evidence supporting this scenario. Rather, PD-1 KO T_ML_ cells displayed a further reduction in their ability to activate key TCR downstream signaling pathways, including NFkB and NFAT. By-passing membrane proximal TCR signaling events restored the activation of key signaling pathways of PD-1-deficient T_ML_ cells. Thus, in the absence of PD-1-mediated inhibition, T_ML_ cells persist but adapt to chronic stimulation by reducing the capacity of the TCR to respond, likely in order to avoid overstimulation. This adaptation may be limited to moderate changes in inhibitory signaling. Indeed, absence of both PD-1 and Lag-3 or of TOX, which controls multiple inhibitory receptors, results in the loss of CD8^+^ T cells (14, 13, 29). Thus, absence of PD-1 may lead to continuous and/or excessive TCR mediated activation, which can be avoided by down tuning TCR signaling. When multiple inhibitory receptors cannot be expressed, down tuning TCR signaling may no longer suffice to avoid continuous/excessive activation, resulting in activation-induced cell death (30).

The findings in chronically stimulated CD8^+^ T cells lacking PD-1 have striking parallels to NK cells that develop in the absence of inhibitory MHC class I ligands or to mature NK cells that are adoptively transferred into an environment lacking MHC class I (31). Persistent engagement of activating receptors in the absence of inhibitory inputs (e.g. via MHC class-I molecules), induces hypo-responsiveness of NK cell activation receptors (32, 33). Although the precise mechanism(s) remain to be elucidated, membrane proximal events, such activation receptor induced as Ca^2+^ flux, are impaired (34). Indeed, intracellular Ca^2+^ mobilization in response to TCR triggering, which is essential for NFAT and full NFkB activation, was reduced in WT T_ML_ cells and did not occur in KO T_ML_ cells. Consistent with a membrane proximal defect, translocation of RelA and NFAT1 in PD-1 KO cells occurred when proximal TCR signaling was bypassed using TNFα and PMA and Ionomycin (P/I), respectively. A key downstream consequence of TCR activation is the production of cytokines. Consistent with impaired TCR signaling, PD-1 KO T_ML_ cells showed reduced constitutive and peptide-induced IFNγ and TNFα transcript levels. Unexpectedly, however, peptide induced cytokine protein expression was not different between PD-1 KO and WT T_ML_ cells. These data raised the possibility that PD-1 limited the translation of cytokine mRNAs in CD8^+^ T cells responding to chronic infection. Similarly, tumor infiltrating CD8^+^ T lymphocytes (TIL) lose their capacity to produce effector cytokines. Importantly, the loss of IFNγ production in TIL is based on post-transcriptional repression (35). PD-1 blockade in a tumor/T cell coculture system improved IFNγ protein production but did not improve IFNγ mRNA levels or stability. Conversely CD28 engagement augmented both IFNγ mRNA stability and protein expression. Thus, the authors speculated that increased IFNγ production upon PD-1 blockade involves increased protein translation, which may not depend on CD28 co-stimulation (35). How the translation block in TIL is regulated is not known. However, IFNγ translation in memory T cells is blocked by the binding of Zfp36l2 to AU-rich elements (AREs) in the 3’ UTR of the *Ifng* mRNAs (36). While similar mechanisms may operate in chronically stimulated CD8^+^ T cells, elucidating the precise basis was beyond the scope of this study and will be addressed in future investigations.

In line with their reduced regeneration in response to acute restimulation, chronic phase PD-1 KO T_ML_ cells expressed reduced levels of the stemness genes *Myb* (24) and *Klf4* (26) compared to WT T_ML_ cells. *Myb* is a PID_AP-1 signature gene and the expression of Jun, which transactivates the human *Myb* promoter via an AP-1-like site (37), is reduced in PD-1 KO compared to WT T_ML_ cells (**Supplementary Table S1**). TCR stimulation of naïve CD8^+^ T cells via the TCR *in vitro* increases *Myb* expression *in vitro* (not depicted) (25). Klf4 is transiently down-regulated in response to acute TCR activation via the TCR activation (not depicted) (27), but is overexpressed in response to chronic TCR stimulation (38). Reduced constitutive TCR signaling in chronically stimulated PD-1 KO T_ML_ cells thus likely accounts for the lower *Myb* and *Klf4* expression. We further addressed the functional importance of reduced *Myb* and *Klf4* expression. Reducing *Myb* expression (2-fold) reduced the generation of differentiated T_EX_ cells, while the generation of T_ML_ cells was not altered. As *Myb*-deficiency prevents the generation of T_ML_ cells during chronic infection (25), a further reduction of *Myb* levels, reproducing the 4-fold reduced expression in PD-1 KO T_ML_ cells, may thus be needed to see effects on T_ML_ cells. Reduced *Klf4* expression prevented the generation of T_ML_ cells, compatible with a role of normal Klf4 levels in the regeneration of T_ML_ cells. *Klf4* together with *Oct4*, *Sox2* and *c-Myc* is used to reprogram mouse somatic cells into pluripotent stem cells (39). Further, Klf4 expression limits the proliferation of CD8^+^ T cells in response to acute TCR triggering *in vitro* (27). In response to tumors, Klf4-deficiency in CD8^+^ T cells reduces the generation of transitory T_EX_ cells without an overt effect on T_ML_ cells (38). Further analyses will thus be needed to address the precise role of *Klf4* in chronic infection. Finally, we noted that PD-1 KO T_ML_ cells included a lower fraction of CD62L^+^ cells, which harbor most of the self-renewal capacity of the T_ML_ cells (25). While this may contribute to the reduced regeneration of T_ML_ cells, the 2-fold reduced fraction of CD62L^+^ cells may not fully account for the 5.7-fold reduced generation of secondary Tcf1^+^ cells in response to recall stimulation.

T_ML_ cells constitutively lacking PD-1 showed reduced regeneration in response to acute restimulation. We further observed that transient PD-1 blockade during the chronic phase of the infection reduced the regeneration of T_ML_ cells. These findings have potential implications for checkpoint blockade immune therapy of cancer. PD-1 blockade expands tumour-specific CD8^+^ T cells and improves their effector functions and this can lead to tumour control depending on the presence of T_ML_ cells (40, 41). However, the beneficial effects are transient and the responding cells fail to show stable improvements (42). Worse, our data raise the possibility that repeated cycles of PD-1 blockade reduces the regeneration of T_ML_ cells, which may progressively limit the effectiveness of checkpoint blockade immune therapy. It may thus be necessary to balance the beneficial effect of generating more effector-like cells, which limit tumour progression, with the negative effect of reducing stemness, which would result in the gradual loss of tumour-specific T cells. This possibility remains to be addressed.

## Materials and methods

### Mice

C57BL/6 (B6) (CD45.2^+^) mice were obtained from Envigo (Gannat, France), CD45.1 congenic B6 mice were bred locally. PD-1 KO (*Pdcd1^-/-^*) mice (43) were provided by T. Honjo, (Kyoto, Japan). P14 TCR transgenic mice (44) were provided by H.-P. Pircher (Freiburg, Germany), Vβ5 TCRβ only transgenic mice (45) were provided by P. Fink (Seattle, USA). *Tcf7*^GFP^ (17) and *Tcf7*^DTR-GFP^ (40) mice have been described. *Tcf7*^GFP^ P14 (CD45.2^+^ or CD45.1/2^+^), *Tcf7*^DTR-GFP^ P14 (CD45.2^+^), PD-1 KO P14 (CD45.2^+^) and PD-1 KO *Tcf7*^GFP^ P14 (CD45.2^+^) mice were obtained by breeding.

Mouse strains were maintained in the SPF animal facility of the University of Lausanne. Both male and female mice between 6 and 12 weeks of age were used for experiments whereby donors and recipients were sex matched for adoptive T cell transfers. Animal experiments were conducted in accordance with protocols approved by the veterinary authorities of the Canton de Vaud.

### LCMV infections and viral titers

The LCMV 53b Armstrong (Arm) and LCMV clone 13 (cl13) strains were propagated in baby hamster kidney cells and titrated on Vero African green monkey kidney cells according to an established protocol (46). Frozen stocks were diluted in PBS. Mice were infected with 2×10^5^ plaque-forming units (PFU) of LCMV Arm (i.p.) or 2×10^6^ PFU of LCMV cl13 (i.v.).

To determine viral titers, kidneys from LCMV-infected mice were frozen at -80°C. Diluted samples were used to infect Vero cells, and viral titers were determined using a LCMV focus-forming assay as described elsewhere (46). LCMV Plaque Forming Units (PFU) were calculated per gram of kidney tissue.

### Anti-PD-L1 and anti-PD-1 treatment

Chronically infected mice were injected i.p. with of 200 µg of rat anti-mouse PD-L1 (10F.9G2) or rat IgG2b isotype control (LTF-2), starting on d20 p.i., using 4 day intervals 5 times and analyzed on d41 p.i. or starting on d24 p.i. 4 day intervals 3 times and analyzed on d36 p.i. During the recall response recipient mice were treated by i.p. with 200 µg of rat anti-mouse PD-1 (RPM1.14) or rat IgG2a (2A3) isotype starting at the time of infection (d0) every 2 days 4 times and analysis on d8.

### CD8^+^ T cell purification and adoptive transfers

Single cell suspensions were obtained by mashing the spleen through a 40 µm nylon strainer (BD Falcon). Red blood cell lysis was performed with a hypotonic ammonium-chloride-potassium (ACK) buffer for 4 min at room temperature (RT) and was stopped by addition of complete DMEM medium. CD8^+^ T cells were purified using mouse CD8^+^ T cell enrichment kit (StemCell Technologies). For chronic infections, purified P14 cells (CD45.2 or CD45.1/2) were adoptively transferred i.v. into naïve Vβ5 (CD45.1) mice one day prior to infection with LCMV cl13 (d-1). Vβ5 hosts were transferred with 250–500 PD-1 KO P14 cells or 500–5000 WT P14 cells, as detailed in the Figure Legends. Alternatively, Vβ5 hosts were transferred with 1:1 mixtures of PD-1 KO P14 (CD45.2) and WT P14 cells (CD45.1/2), 500 cells total. For acute infections, 10^4^ purified P14 cells were adoptively transferred i.v. into naïve B6 host mice one day prior to infection with LCMV Armstrong (d-1). For recall responses, ~10^4^ flow sorted Ly108^+^ Tim3^-^, Ly108^-^ or *Tcf7*^GFP+^ P14 cells were transferred into naïve secondary Vβ5 or B6 recipients that were infected on the same day with LCMV Arm (d0).

### Plasmids, Lentivirus production and CD8^+^ T cell transduction

Lentivirus (LV) U6-shRNA hPGK-mCherry constructs specific for *Tcf7*, *Myb* and *Klf4* were obtained from VectorBuilder (see Key Resource Table). 2^nd^ generation packaging constructs (pCMV-dR8–74 and pMD2-G) were provided by D. Trono (EPFL, Lausanne).

For LV production, 293T cells were transiently transfected with knockdown and packaging plasmids (pCMV-dR8.74 and pMD2.G) using lipofectamine 2000 (ThermoFisher) in the absence of antibiotics. 48h after transfection, culture supernatants were collected, filtered (0.45 µM Millex) and either used directly to transduce activated CD8^+^ T cells or stored at -80°C.

For LV transduction, CD8^+^ T cells were purified from the spleen of naïve P14 or *Tcf7*^DTR-GFP^ P14 mice (herein referred to as *Tcf7*^GFP^). Purified cells were activated with Dynabeads Mouse T-Activator CD3/CD28 (ThermoFisher) (cell:beads ratio of 1:1) in the presence of recombinant human IL2 (50 ng/mL) (gift from N. Rufer, CHUV) for 24 h *in vitro* before the addition of viral supernatants. LV transduction was performed using spin infection (1800 rpm for 90 min at 30°C) in the presence of polybrene (4 µg/mL) (Sigma). The cells were cultured overnight at 37°C and 3x10^4^ P14 cells were adoptively transferred into Vβ5 (CD45.1) mice that had been infected with LCMV cl 13 one day before. Separately, P14 cells were kept in culture for 48 h to determine the transduction efficiency based on Cherry expression.

### Flow cytometry and cell sorting

Spleens were mashed through a 40 µM nylon cell strainer to obtain single cell suspensions and red blood cell were lysed using ACK buffer.

Surface staining was performed using mAbs (using the reagents listed in the Key Resource Table). diluted in PBS supplemented with 2% FCS for 20’ at 4°C. Cells were washed twice before the addition of secondary Abs together with Zombie Aqua Fixable Viability kit (BioLegend). For apoptosis assays, total splenocytes were cultured for 4h at 37°C without growth factors. The cells were surface stained, followed by Annexin V-APC staining (Apoptosis Detection Kit, eBioscience). 7-AAD was added 10’ prior to the acquisition at the flow cytometer.

For nuclear staining including cell cycle analysis, cells were first surface stained and subsequently fixed using the FoxP3 transcription factor staining buffer set (eBioscience: cat # 00-5523). For nuclear staining, Abs were diluted in 1x Permeabilization Buffer (Perm buffer) and incubated for 1h at 4°C. Cells were washed twice with 1x Perm buffer before incubation with secondary Abs diluted in 1x Perm buffer for 15’ at 4°C. For cell cycle analysis cells, Ki67-FITC was diluted in 1x Perm buffer and incubated for 1h at 4°C. After washing, DAPI (2 µg/mL) diluted in 1x Perm buffer was added for 10’ at 4°C.

For cytokine production, splenocytes were re-stimulated *in vitro* with 1 µM LCMV gp33-41 (gp33) peptide for 5h at 37°C. Brefeldin A (5 µg/mL) was added after 30’ of incubation. For intracellular staining, the cells were surface stained, followed by fixation and permeabilization (Intracellular Fixation & Permeabilization Buffer Set, eBioscience kit: Cat # 88-8824).

For phospho flow, purified CD8^+^ T cells harbouring P14 cells were stimulated *in vitro* with 1 µM gp33 peptide for 2h or 4h at 37°C in the presence of splenocytes from naïve mice. Cells were directly fixed with BD Cytokix (BD Bioscience, Cat # 554655) solution for 20 min at 37°C and washed with PBS containing 2% FCS. A Fc-block step was performed using 24G2 supernatant for 5 min at 4°C followed by surface staining for 20 min at 4°C. Cells were washed and permeabilized in BD Perm buffer III (BD Bioscience, cat # 558050) for 30 min at 4°C. After washing, antibodies for phosphorylated Erk (Phospho-p44/42 MAPK (Erk1/2) (Thr202/Tyr204, Clone D13.14.4E) diluted in PBS 2% FCS, were added for 1h at room temperature (rt) in the dark, followed by addition of secondary antibodies for 15 min at room temperature in the dark. Cells were washed in PBS and resuspended in PBS 2% FCS for acquisition at the flow cytometer.

For Ca^2+^ flux analysis, purified CD8^+^ T cells harbouring P14 cells were incubated with Indo-1 (ThermoFisher, 2 uM final) for 30 min at 37°C and washed twice in RPMI 10% FCS before surface staining with anti-CD45.2 in PBS 2% FCS for 15 min at 4°C. After washing, cells were resuspended in RPMI 10% FCS and either not activated (no) or activated for 30 sec by gp33 tetramer (3ug/ml) or Ionomycin (10ug/ml) addition before cell acquisition for 1–3 min for each condition.

Data acquisition was performed on LSR-II or Fortessa flow cytometers (BD). Data were analysed with FlowJo (TreeStar).

For cell sorting, P14 cells (CD45.2) were enriched with the mouse CD8^+^ T cell enrichment kit (StemCell Technologies) and then stained for CD45.1 (Vβ5 host) and CD45.2 (P14). Ly108^+^ Tim3^-^ and Ly108^-^ Tim3^+^ or *Tcf7*^GFP+^ and *Tcf7*^GFP-^ cells were flow sorted on either FACSAriaIII (BD) or FACSAriaIIU (BD) flow cytometer. The purity of sorted cells was greater than 99%, assessed by post-sort analysis.

### Imaging flow cytometry

Flow sorted WT or PD-1 KO P14 *Tcf7*^GFP^ cells at d28 post LCMV cl13 infection (WT cl13) and (PD-1 KO cl13) were restimulated *in vitro* with plate bound anti-CD3 and anit-CD28 mAbs (4ug/ml each) for 4 h or were cultured without activation (rested).

Cells were fixed in 2% paraformaldehyde and permeabilized using 0.1% Triton X-100 (Sigma) for 10 min at room temperature (rt), and stained for NFAT1 or NF-kB p65 (Cell Signaling) for 20 min at rt before washing and incubation with secondary Abs. The DNA dye DAPI (0.5 ug/ml; ThermoFisher) was added and incubated overnight before data acquisition. Images from at least 6000 events per condition were obtained using Amnis Imagestream.

For data analyses, DAPI staining was used to define the nucleus and colocalization with RelA or NFAT1 was estimated by similarity (IDEAS software, Amnis). Nuclear localization was defined by a positive similarity score, representing the relative colocalization of the nuclear dye DAPI and the NFAT1 or NF-kB p65. Most rested P14 cells had a negative similarity score, indicating the absence of nuclear NFAT1 and NF-kB. Nuclear translocation in response to stimulation was estimated as the percent of cells with a similarity score >1 minus the percent of rested cells with a similarity score >1.

### RT-qPCR analysis

For the detection of *Tcf7*, *c-Myb* and *Klf4*, T_N_, d8 or d36 Ly108^+^ or Tcf7^GFP+^ and d8 or d36 Ly108^-^ or Tcf7^GFP-^ P14 cells were flow sorted and lysed with Trizol LS (Life Technologies) and RNA was extracted using Direct-zol™ RNA MiniPrep kit (Zymo Research). cDNA was synthesised with the SuperScript III First-Strand Synthesis System (ThermoFisher Scientific). KAPA SYBR FAST qPCR Kit master Mix (Kapabiosystems) was used to perform real-time quantitative PCR on a LightCycler 480 Instrument (Roche), using the primers indicated in the Key Resource Table.

### Bulk RNAseq analysis

CD62L^+^ CD8^+^ T cells form naïve wild type (WT) P14 or PD-1 KO P14 mice (CD45.2) were flow-sorted to obtain cellular RNA or for transfers into Vβ5 hosts (CD45.1). Recipient mice were infected with LCMV cl13 the following day. Some Vβ5 hosts were injected with anti-PD-L1 (200 µg B7-H1; clone 10F.9G2) at d24, d28 and d32 post infection. Thirty-six days after infection, splenic Ly108^+^ Tim3^-^ and Ly108^-^ P14 cells from WT and PD-1 KO mice were flow-sorted. Sorted cells were lysed and stored in Trizol before extraction of total cellular RNA using the Direct-zol™ RNA MiniPrep kit (Zymo Research).

RNA quality was assessed on a Fragment Analyzer (Agilent Technologies) and all RNAs had an RQN ≥ 8.7. From 50 ng total RNA, mRNA was isolated with the NEBNext Poly(A) mRNA Magnetic Isolation Module. RNA-seq libraries were then prepared from the mRNA using the NEBNext Ultra II Directional RNA Library Prep Kit for Illumina (New England Biolabs, Massachusetts, USA). Libraries were quantified by a fluorimetric method, and their quality assessed on a Fragment Analyzer (Agilent Technologies). Cluster generation was performed with the resulting libraries using Illumina HiSeq 3000/4000 SR Cluster Kit reagents. Libraries were sequenced on the Illumina HiSeq 4000 with HiSeq 3000/4000 SBS Kit reagents for 150 cycles. Sequencing data were demultiplexed with the bcl2fastq Conversion Software (v. 2.20, Illumina; San Diego, California, USA).

Purity-filtered reads were trimmed to remove adapters and low-quality bases with Cutadapt [v. 1.8 (47)]. Reads matching to ribosomal RNA sequences were removed with fastq_screen (v. 0.11.1). The remaining reads were further filtered to remove low complexity reads with reaper [v. 15-065 (48)]. Reads were aligned against the *Mus musculus* (GRCm38.92) genome using STAR [v. 2.5.3a (49)]. The number of read counts per gene locus was summarized with htseq-count [v. 0.9.1 (50)] using the *Mus musculus* (GRCm38.92) gene annotation. Quality of the RNA-seq data alignment was assessed using RSeQC [v. 2.3.7 (51)].

Differential gene expression analysis was performed using R (v. 3.5.3) (52). Genes were filtered to retain only the ones detected at 1 count per million (cpm) in at least 1 sample, yielding 13,303 retained genes. Normalization factors were calculated using the weighted trimmed mean of M-values (TMM) method implemented in the edgeR package (v. 3.24.3) (53). Gene counts were next transformed to log_2_(cpm) and the mean-variance modeling was performed using the voom function implemented in the limma package (v. 3.38.3) (54, 55). A linear model was fitted for each gene, and moderated t-statistics were computed using the lmFit and eBayes functions of the limma package (56). Genes differentially expressed between pairwise comparisons were considered significant at adjusted p-value<0.05 after Benjamini-Hochberg (BH) adjustment (57).

Gene set enrichment analysis (GSEA) was performed using the clusterProfiler package [v. 4.0.4 (58)] in R v. 4.1.0, against the Hallmark and the Pathway Interaction Database (PID) (59) gene sets available on the Molecular Signatures Database [MSigDB, v.7.4 (60)]. The genes were sorted according to their t-statistic and analysed for enrichment using the GSEA function of the clusterProfiler package, with a seed set to 1234 and a minimum p-value boundary of 1e-50. Enriched gene sets were considered significant at adjusted p-value<0.05 after BH adjustment.

### Single cell RNA sequencing analysis

CD62L^+^ CD8^+^ T cells form naïve WT P14 or PD-1 KO mice (CD45.2) were flow sorted and transferred into Vβ5 hosts (CD45.1), which were infected with LCMV cl13 one day later. Sorted WT P14 cells were also transferred into B6 hosts (CD45.1), which were infected with LCMV Arm one day later. On day 28 post infection, P14 cells were flow sorted and either rested for 4h or restimulated *in vitro* with plate bound anti-CD3/28 antibodies for 4 h and then subjected to scRNAseq analysis.

Cell lysis and RNA capture was performed according to the 10x Genomics protocol (Single Cell 3’ v3 chemistry). The cDNA libraries were generated according to the manufacturer’s protocol (Illumina) and further sequenced (paired-end) with NovaSeq 6000 technology (Illumina).

The raw sequencing reads were filtered, demultiplexed and aligned to the mouse genome (mm10) using the 10x Genomics Cell Ranger pipeline (version 6.0.2). The Cell Ranger aggr function was used to combine the counts of all called cell from all samples into a single UMI count. Subsequently, the raw count matrix was imported into R (v.4.1.2) and scRNAseq data was analyzed using the Seurat package (v.4.3.0) for R (61).

The raw count matrix was filtered to only retain cells that had between 500 and 5001 genes expressed, less than 50000 total UMI counts and less than 10% mitochondrial gene expression (n=32665). The median number of genes detected per cell was n=1484 for WT P14 cells LCMV Arm (WT Arm) rested, n=1614 for WT Arm aCD3/28 stimulated, n=1331 for WT cl13 rested, n=1188 for WT cl13 aCD3/28, n=1477 for PD-1 KO rested, and n=1448 for PD-1 KO aCD3/28 cells.

The filtered raw counts were log-normalized (to 10000 transcripts per cell) and scaled using the NormalizeData and ScaleData functions implemented in the Seurat package. The top 2000 most variable genes were selected to compute principal components. Cell clustering integrating all conditions was performed using the shared nearest neighbor (SNN) modularity optimization-based algorithm implemented in the FindNeighbors and FindClusters functions, with 25 principal components and a resolution parameter of 0.4. The clustering resulted in a total of 11 clusters, with clusters C1 to C9 encompassing 96.5% of all cells.

Genes differentially expressed between unstimulated and restimulated cells from the same sample or between specific pairs of clusters were determined using the FindMarkers function of the Seurat package, using default parameters (i.e. Wilcoxon test and Bonferroni correction of *p*-values). To determine whether differentially expressed genes were linked to pathways or gene sets, we performed gene set over-representation analysis using the enricher function of the clusterProfiler package (v.4.2.2), testing up-regulated and down-regulated genes separately, and adjusting p-values using the Benjamini-Hochberg (BH) procedure (57). Lists of gene sets were obtained from the Molecular Signature Database (MSigDB) (60) using the msigdbr package (v.7.5.1), namely the Hallmark gene sets (62) and the Pathway interaction database (PID) gene sets.

Differentially expressed genes obtained in both the bulk and the single-cell RNA sequencing datasets were tested for over-representation of an exhaustion signature defined following a method similar to (20). Affymetrix array data of exhausted (day 22–35 chronic infection GP33-specific) and effector (day 8 acute infection GP33-specific) T cells published by (11) was downloaded from the Gene Expression Omnibus (GSE9650). We computed the RMA (Robust Multi-array Average) expression measure using the rma function of the affy package (v.1.64.0) and calculated the log_2_(fold change) of each probe in exhausted versus effector cells. The exhaustion signature included all genes with at least one probe with log_2_(fold change) higher than 1 (n=130 genes up-regulated in exhausted T cells).

### Quantification and statistical analysis

Fold-expansion of P14 T cells was determined relative to an estimated 10% of engraftment of the transferred cells (63).

All statistical analyses were performed using the latest version of GraphPad Prism between 8.0 and 9.1.2 (GraphPad Software). Statistical significance was achieved when p values were <0.05 with a 95% confidence level (*=p<0.05, **=p<0.01, ***=p<0.001, ****=p<0.001) as indicated with asterisks in the figures; p>0.5 was considered non-significant (ns). Data are depicted in mean ± SD. Unpaired *t* test (two-tailed, 95% confidence level) was used to compare 2 data sets. Paired *t* test (two-tailed, 95% confidence level) was used to compare paired values, e.g. WT and PD-1 KO values that were derived from the same host. ANOVA was used to compare >2 groups.

## Data Availability

Sequencing data generated in this study have been deposited into the GEO database with accession number GSE277648 (bulk RNAseq) and GSE277649 (scRNAseq).

## Supplementary material

Supplementary Table 1 Bulk RNAseq: Genes differentially expressed between the indicated populations of PD-1 KO and WT P14 cells. Related to **Figure 1** and **Figure 6**.

Supplementary Table 2 Bulk RNAseq: Pathway analyses of genes differentially expressed between the indicated populations of PD-1 KO and WT P14 cells. Related to **Figure 1** and **Figure 6**.

Supplementary Table 3 ScRNAseq: Number of cells per sample and cluster. Related to **Figure 2**.

Supplementary Table 4 ScRNAseq: Genes differentially expressed between the indicated subsets of activated PD-1 KO versus WT P14 cells. Related to **Figure 2**.

Supplementary Table 5 ScRNAseq: Pathway analyses of genes differentially expressed between the indicated subsets of PD-1 KO versus WT P14 subsets. Related to **Figure 2**.
