# Supplementary material for: Memory-like CD8+ T cells lacking PD-1 adapt to persistent stimulation by reducing TCR signal transduction rather than increasing exhaustion
Source: Front Immunol. 2026 Feb 5;17:1743170. doi: 10.3389/fimmu.2026.1743170 (PMC12916634; doi:10.3389/fimmu.2026.1743170)
Supplement: Supplementary file 6 [file DataSheet1.pdf]

Supplementary Material

1 Supplementary Data

2 Supplementary Figures and Tables

2.1 Supplementary Figures

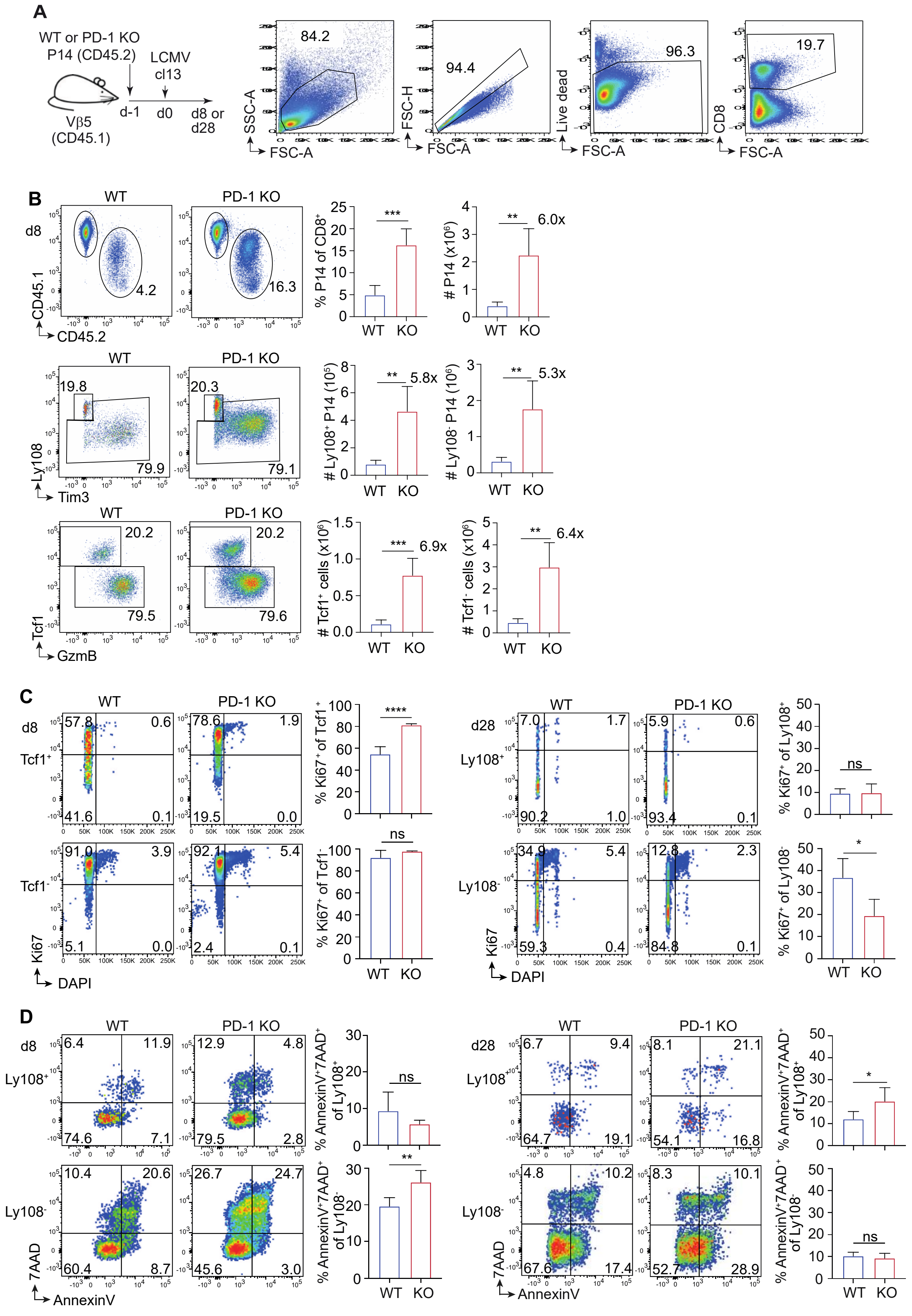
A
WT or PD-1 KO
P14 (CD45.2)
LCMV
cl13
Vβ5
(CD45.1)
d-1
d0
d8 or
d28
84.2
94.4
96.3
19.7
SSC-A
FSC-A
FSC-H
Live dead
CD8
B
WT
PD-1 KO
d8
CD45.1
CD45.2
4.2
16.3
% P14 of CD8+
# P14 (x10^6)
***
**
6.0x
WT
KO
19.8
79.9
20.3
79.1
Ly108
Tim3
Ly108+ P14 (10^5)
Ly108- P14 (10^6)
5.8x
5.3x
20.2
79.5
79.6
Tcf1
GzmB
Tcf1+ cells (x10^6)
Tcf1- cells (x10^6)
6.9x
6.4x
C
Tcf1+
Tcf1-
Ki67
DAPI
57.8
0.6
41.6
0.1
78.6
1.9
19.5
0.0
91.0
3.9
5.1
0.0
92.1
5.4
2.4
0.1
% Ki67+ of Tcf1+
% Ki67+ of Tcf1-
****
ns
d28
Ly108+
Ly108-
7.0
1.7
90.2
1.0
5.9
0.6
93.4
0.1
34.9
5.4
59.3
0.4
12.8
2.3
84.8
0.1
% Ki67+ of Ly108+
% Ki67+ of Ly108-
*
D
6.4
11.9
74.6
7.1
12.9
4.8
79.5
2.8
10.4
20.6
60.4
8.7
26.7
24.7
45.6
3.0
7AAD
AnnexinV
% AnnexinV+7AAD+ of Ly108+
% AnnexinV+7AAD+ of Ly108-
6.7
9.4
64.7
19.1
8.1
21.1
54.1
16.8
4.8
10.2
67.6
17.4
8.3
10.1
52.7
28.9

Supplementary Figure 1: Presence of PD-1 KO T_{ML} cells during chronic infection.
(**A**) Experimental schematic: Naïve wild-type or *Pdcd1*$^{-/-}$ (PD-1 KO) P14 cells (CD45.2) were adoptively transferred into congenically distinct TCRβ (Vβ5) transgenic B6 mice (CD45.1) that were infected one day later with LCMV cl13. On the indicated day of infection, P14 cells present in the spleen were characterized using flow cytometry. Live cells were gated using FSC-A and SSC-A, followed by doublet discrimination using FSC-H/FSC-A. Next, singlets were gated for alive cells, $CD8^+$ and congenic markers CD45.1 and CD45.2 were used to gate P14 cells. **(B, D)** Equal numbers of naïve WT or PD-1 KO P14 cells (CD45.2) (500 cells each) or (**C**) unequal numbers of naïve WT or PD-1 KO P14 cells (CD45.2) (5-fold more WT cells) were injected into Vβ5 mice (CD45.1), which were then infected with LCMV cl13 and analyzed at d28 post infection (p.i.). **(B)** Gated P14 cells ($CD45.2^+$) were analyzed for the presence of $Tcf1^+$ (T_{ML}) versus $Tcf1^-$ (T_{EX}) cells at d 8 p.i.. (**C**) Gated $Tcf1^+$ and $Tcf1^-$ P14 cells ($CD45.2^+$) at d8 and d28 p.i. were analyzed for cycling cells ($Ki67^+$ and DAPI) **(D)** Total splenocytes at d8 or d28 p.i. were cultured for 4h in the absence of growth factors and gated $Ly108^+$ (T_{ML}) and $Ly108^-$ (T_{EX}) P14 cells ($CD45.2^+$) were analyzed for 7-AAD uptake and Annexin-V staining. Data in **(A-D)** derive from n=4-5 mice per group and are representative of n=3-4 independent experiments. All bar graphs show means ±SD. Statistics; Unpaired two-tailed t-test. Significance (*p<0.05, **p<0.01, ***p<0.001, **** p<0.0001, ns=not significant p>0.05).

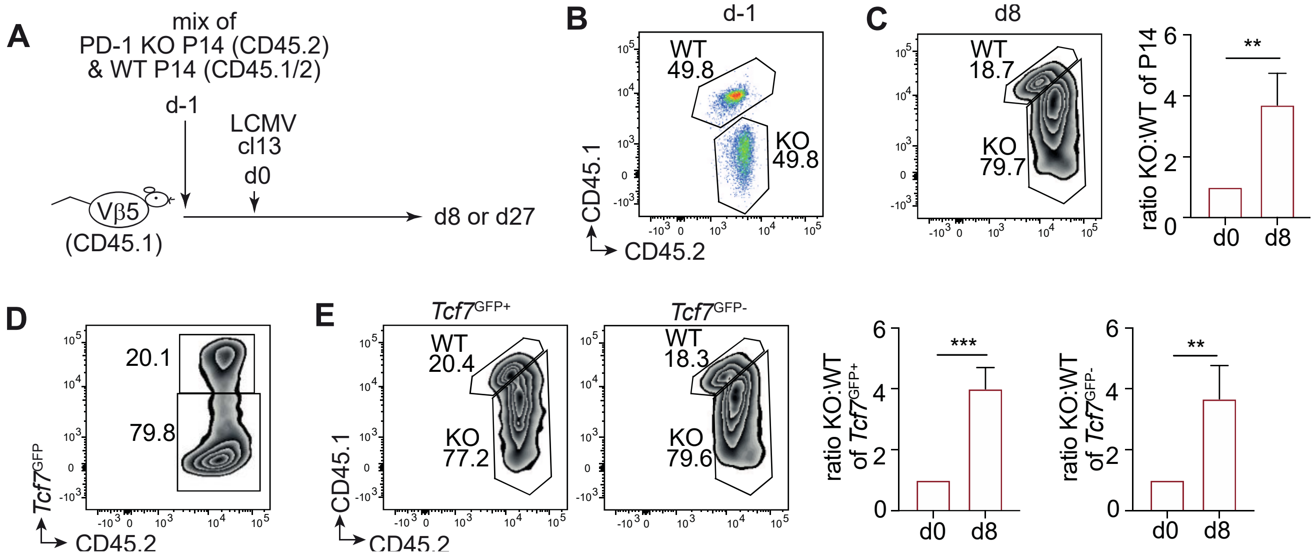

Supplementary Figure 2: Analysis of PD-1 KO T_{ML} cells in co-transfer experiments.
(**A-E**) PD-1 KO (CD45.2) and WT *Tcf7*$^{GFP+}$ P14 (CD45.1/2) cells were mixed and transferred into Vβ5 mice (CD45.1) that were then infected with LCMV cl13 and analyzed on d8. Gated P14 cells were analyzed for the contribution of PD-1 KO cells (CD45.2) at (**B**) input (d-1) and (**C**) at d8 p.i.. (**D**) P14 cells were analyzed for *Tcf7*$^{GFP+}$ expression and gated *Tcf7*$^{GFP+}$ (T_{ML}) and *Tcf7*$^{GFP-}$ P14 cells (T_{EX}) were analyzed for the contribution of PD-1 KO cells (CD45.2) on d8 (**E**).
Data in (**B-E**) derive from 4-5 mice per group and are representative of n=3-4 independent experiments. Bar graphs show the mean percentage (±SD). Paired t-test was performed to determine statistically significant differences (*p<0.05, **p<0.01, ***p<0.001, ns=not significant p>0.05).

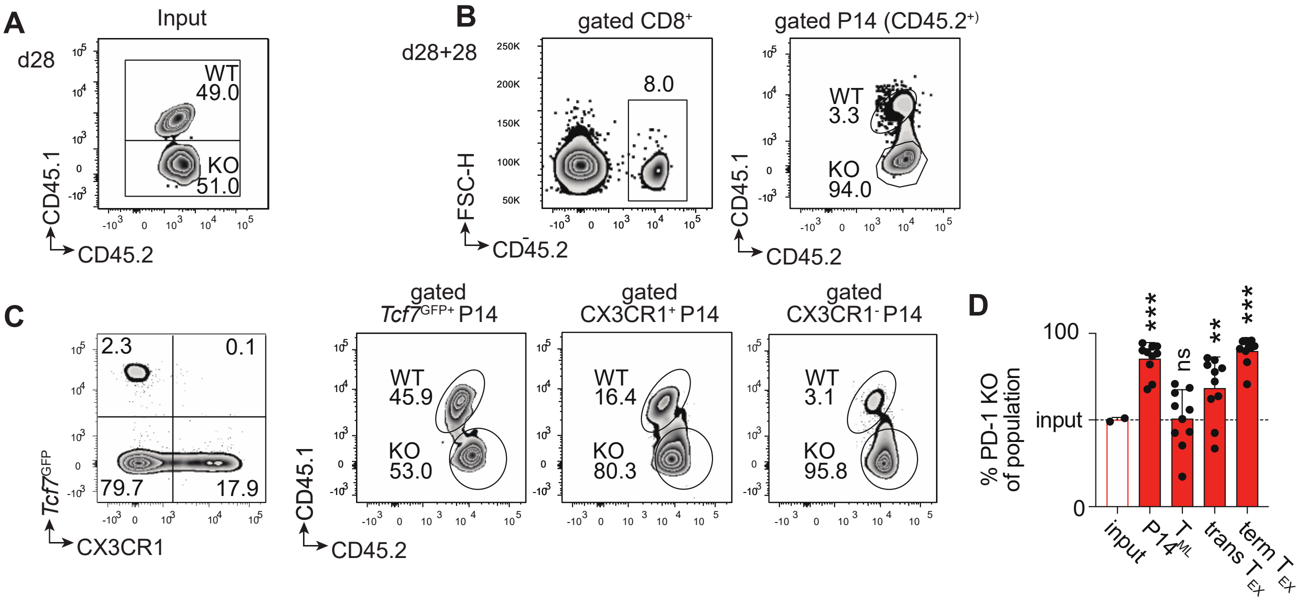

Supplementary Figure 3: Analysis of PD-1 KO T_{ML} cells in retransfer experiments.
(**A-D**) PD-1 KO *Tcf7*$^{GFP+}$ (CD45.2) or WT *Tcf7*$^{GFP+}$ P14 (CD45.1/2) cells transferred into Vβ5 mice (CD45.1) that were then infected with LCMV cl13. Twenty-eight days later WT and PD-1 KO T_{ML} cells were flow sorted, mixed (**A**), transferred into LCMV cl13 infection time matched Vβ5 recipients. Secondary recipients were analyzed 28 days later (d28+28). (**B**) Gated P14 cells were analyzed for the contribution of PD-1 KO cells (CD45.2). (**C, D**) P14 cells were analyzed for the expression of *Tcf7*^{GFP} versus Cx3cr1 and the T_{ML} (*Tcf7*$^{GFP+}$ Cx3cr1^{-}), trans T_{EX} (*Tcf7*$^{GFP-}$Cx3cr1^{+}) and term T_{EX} (*Tcf7*$^{GFP-}$ Cx3cr1^{-}) compartments were analyzed for the contribution of PD-1 KO cells. Data are compiled from 2 independent experiments each with n=5 mice per group. Bar graphs show means ±SD. One sided t-test (relative to input) was performed to determine statistically significant differences (*p<0.05, **p<0.01, ***p<0.001, ns=not significant p>0.05).

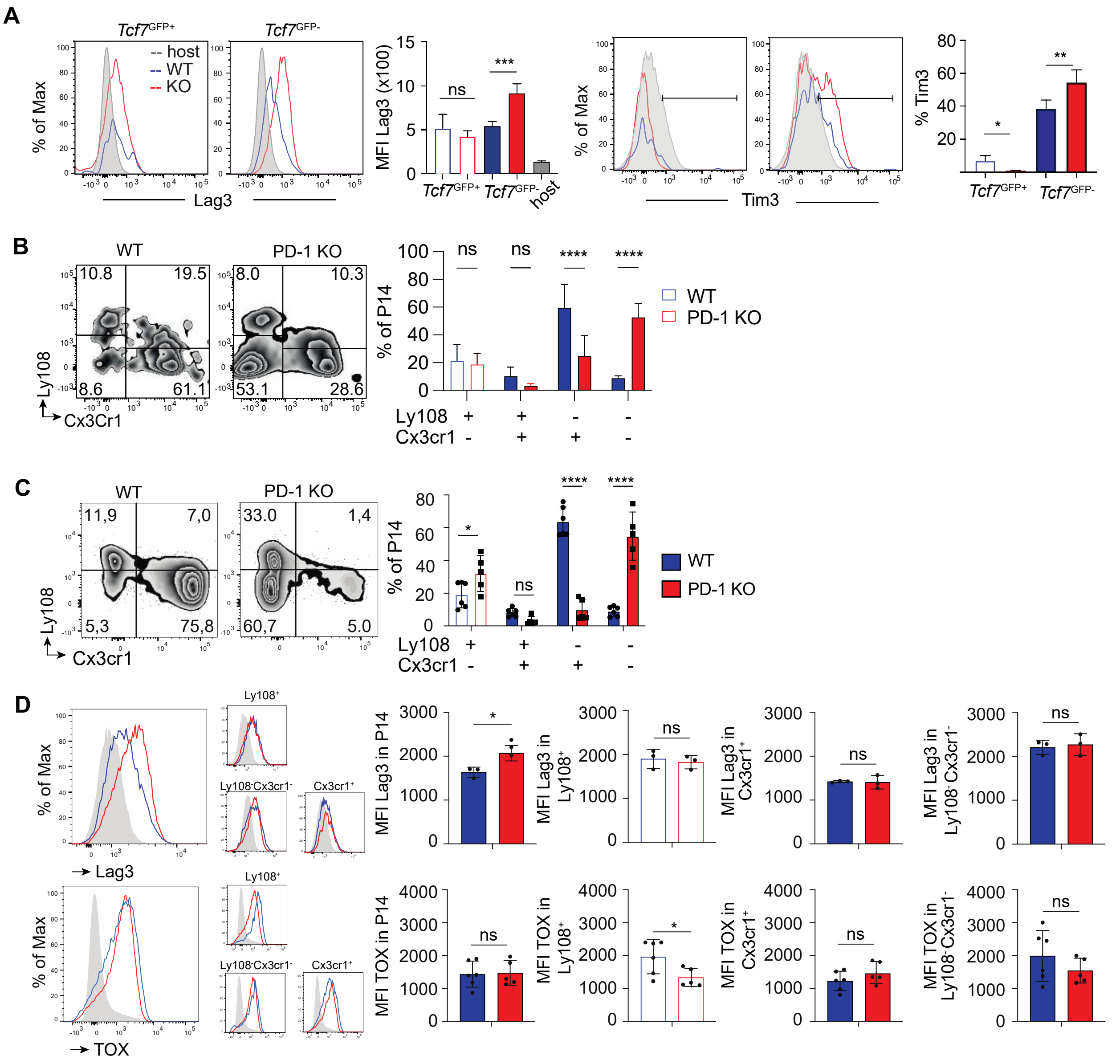

Supplementary Figure 4: Presence of PD-1 KO TML cells during chronic infection.
(**A, B**) PD-1 KO (CD45.2) and WT *Tcf7*$^{GFP+}$ P14 (CD45.1/2) cells were mixed (250 cells each) and transferred into Vβ5 mice (CD45.1) that were then infected with LCMV cl13 and analyzed 28 days later. Gated PD-1 KO and WT TML and TEX cells were analyzed (**A)** for the expression of Lag3 and Tim3 and (**B**) for the expression of Ly108 versus Cx3cr1. **(C, D)** WT P14 (5000) or PD-1 KO P14 cells (CD45.2) (500) were transferred into Vβ5 mice (CD45.1) that were then infected with LCMV cl13 and analyzed 28 days later. (**C**) Gated PD-1 KO and WT TML and TEX cells were analyzed for the of Ly108 versus Cx3cr1. (**D**) Gated TML, transitory TEX and terminal TEX cells were analyzed for the expression of Lag3 and TOX. Data in **(A-D)** derive from n=4-5 mice per group and are representative of n=3-5 independent experiments. All bar graphs show means ±SD. Statistics; Unpaired two-tailed students t-test. Significance (*p<0.05, **p<0.01, ***p<0.001, **** p<0.0001, ns=not significant p>0.05).

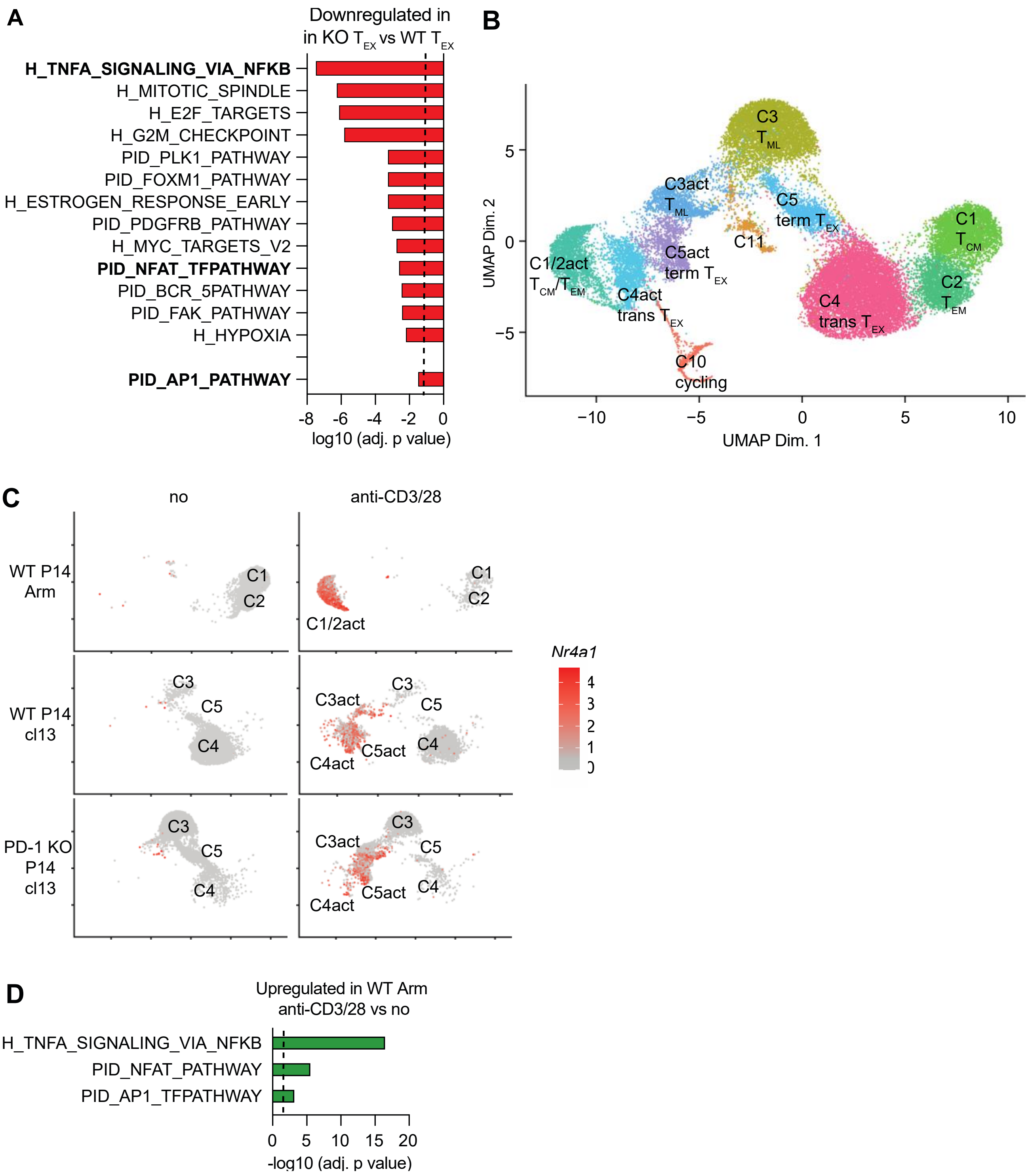

Supplementary Figure 5: Reduced TCR-mediated induction of NFAT and NFkB gene signatures in PD-1 KO T_{ML} cells.

(**A**) Genes differentially expressed between PD-1 KO versus WT T_{EX} cells were subjected to gene set enrichment analysis (GSEA) using the Pathway Interaction Database (PID) and Hallmark (H) database. The bar graph depicts pathways downregulated in PD-1 KO vs WT T_{EX} cells. The broken line indicates the limit of statistical significance (log_{10}(adjusted p-value))=1.3 i.e. $p<0.05$). (**B**) WT and PD-1 KO

P14 cells (d28 post LCMV cl13 infection) or conventional memory P14 cells (d28 post LCMV Arm infection) were flow sorted and rested (no) or restimulated with CD3/28 antibodies *in vitro* for 4 h and then subjected to scRNAseq analysis. UMAP projections of rested and activated cells colored according to their cluster annotation, as assigned based on the expression of key markers genes (see Fig. 2D): LCMV Arm immune cells: T_{CM} (C1) and T_{EM} (C2). LCMV cl13 response: T_{ML} (C3), transitory T_{EX} (trans T_{EX}) (C4) and terminal T_{EX} (term T_{EX}) (C5). C10 which contained cycling cells (mKi67^{+}) and C11, which included <500 cells were excluded from further analysis. The new clusters arising in response to CD3/28 stimulation, that showed signs of activation (*Nr4a1*$^{+}$) and that corresponded to the above subsets that are referred as "act". (**C**) UMAP projection split per library showing the expression of *Nr4a1* per cell. (**D**) Genes differentially expressed in rested versus restimulated P14 cells derived from LCMV Arm immune mice were subjected to over-representation analysis and analyzed for the enrichment of the TNF_NFkB, NFAT and AP-1 gene signatures. The broken line indicates the limit of statistical significance ($-\log_{10}$(adjusted p-value) = 1.3 i.e. $p<0.05$).
(A) Data derive from a single experiment with two biological replicates per population. Data in **(B-D)** derive from a single experiment. The number of cells analyzed per library and cluster is indicated in **Table S3.**

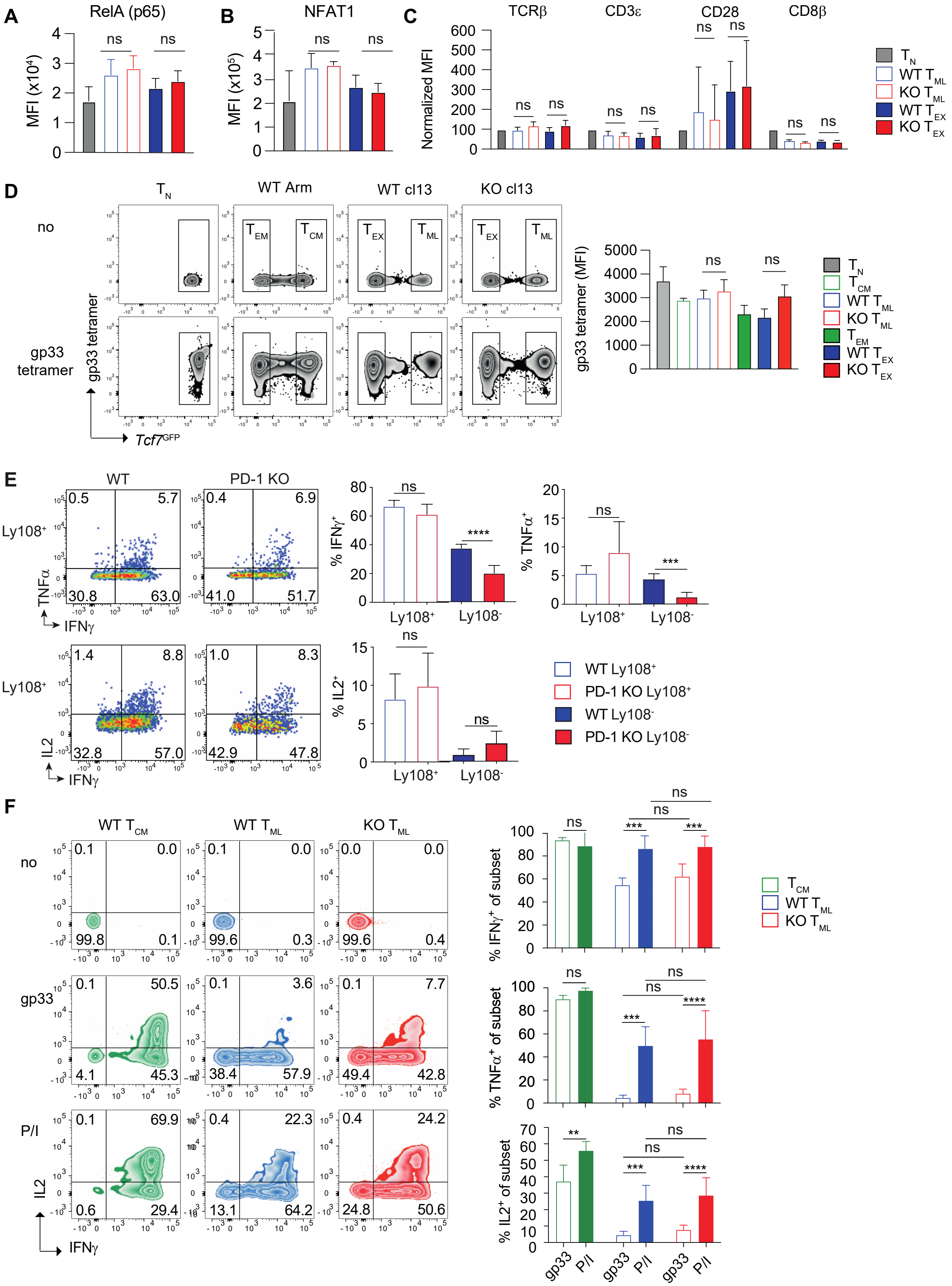
A
RelA (p65)
MFI (x10^4)
ns
B
NFAT1
MFI (x10^5)
C
TCRβ
CD3ε
CD28
CD8β
Normalized MFI
T_N
WT T_ML
KO T_ML
WT T_EX
KO T_EX
D
T_N
WT Arm
WT cl13
KO cl13
no
gp33 tetramer
T_EM
T_CM
T_EX
T_ML
gp33 tetramer
Tcf7^GFP
gp33 tetramer (MFI)
E
WT
PD-1 KO
Ly108+
TNFα
IFNγ
IL2
% IFNγ+
% TNFα+
% IL2+
Ly108+
Ly108-
WT Ly108+
PD-1 KO Ly108+
WT Ly108-
PD-1 KO Ly108-
F
WT T_CM
WT T_ML
KO T_ML
no
gp33
P/I
% IFNγ+ of subset
% TNFα+ of subset
% IL2+ of subset
T_CM
gp33
P/I

Supplementary Figure 6: Impaired activation of NFkB and NFAT in PD-1 KO T_{ML} cells.
(**A**) Expression of RelA and (**B**) NFAT1 by gated PD-1 KO and WT T_{ML} and T_{EX} cells based on imaging flow cytometry. (**C, D**) Gated PD-1 KO and WT T_{ML} and T_{EX} cells were analyzed for (**C**) the expression of TCRβ, CD3ε, CD28 and CD8β or (**D**) for gp33 tetramer binding. The bar graph depicts the mean fluorescent intensity (MFI) of staining (in (**C**) normalized to that of naïve $CD8^+$ T cells). **(E)** WT or PD-1 KO P14 cells (CD45.2) (500 cells each) were transferred into Vβ5 mice (CD45.1), which were then infected with LCMV cl13. At d28 post infection splenocytes were restimulated with gp33 peptide and gated $Ly108^+$ (T_{ML}) and $Ly108^-$ cells (T_{EX}) were analyzed for the production of IFNγ, TNFα and IL2. (**F**) Total $CD8^+$ T cells containing WT or PD-1 KO P14 cells were purified from the spleen of LCMV cl13 infected Vβ5 mice or LCMV Arm infected WT mice at d28 post infection were left unstimulated (no), re-stimulated with gp33 peptide (gp33) or with PMA/Ionomycin (P/I) for 5h. Gated WT T_{ML} and PD-1 KO T_{ML} cells ($Ly108^+$) were analyzed for the production of IFNγ, TNFα and IL2. Data in (**A, B**) are single determinations compiled from n=2-3 independent experiments. Data in (**C**) are compiled from n=2 independent experiments with a total of n=3-5 determinations. Data in (**D**) are compiled from n=4 independent experiments with a total of n=4-6 determinations. Data in **(E)** derive from n=4-5 mice per group and are representative of n=3-4 independent experiments. Data in (**F**) derive from a single experiment with n=5-6 mice per group and are representative of n=3 independent experiments. All bar graphs show means ±SD. Statistics; **(A-E)** Unpaired two-tailed students t-test between the indicated WT and PD-1 KO populations. **(F)** Two-way ANOVA whereby only selected comparisons are shown. Significance (*p<0.05, **p<0.01, ***p<0.001, **** p<0.0001, ns=not significant p>0.05).

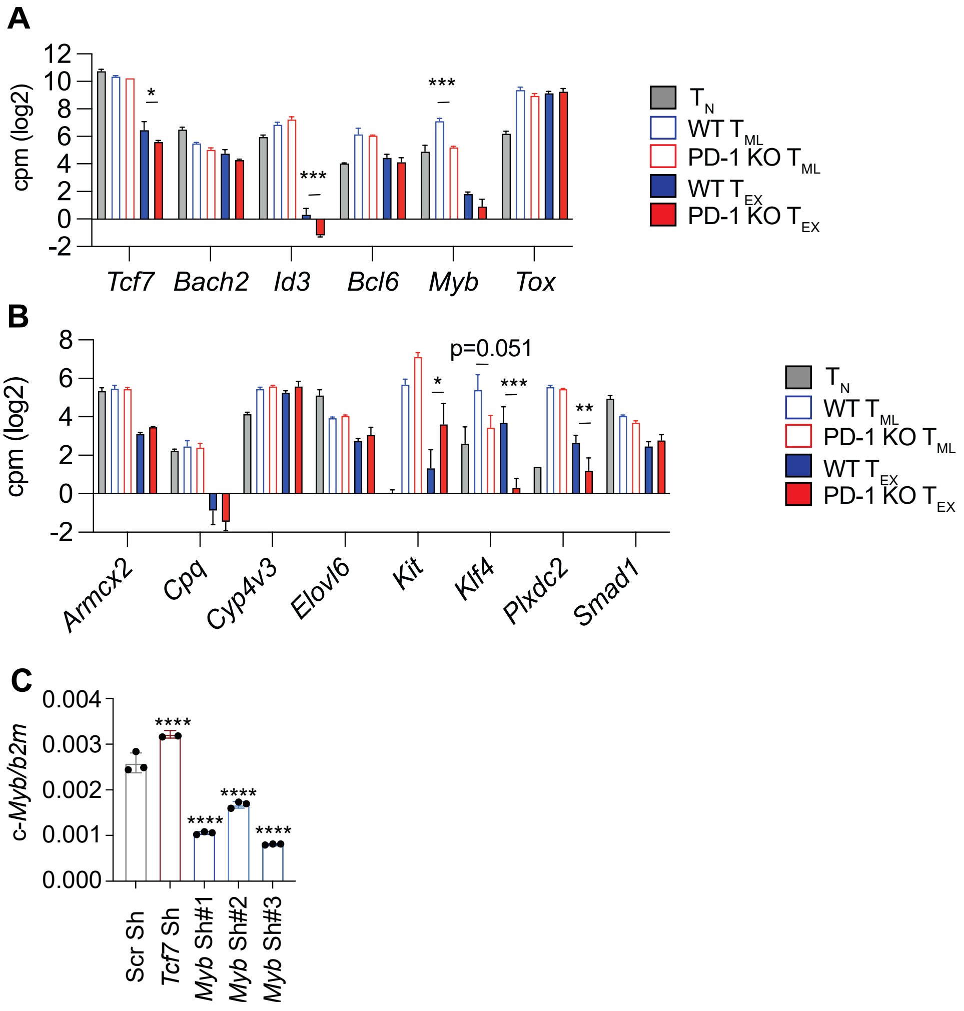

Supplementary Figure 7: Basis for the reduced stemness of PD-1 KO T_{ML} cells.
(**A**) Expression of transcription factors involved in $CD8^+$ T cells stemness in WT and PD-1 KO T_{ML} and T_{EX} cells based on bulk RNAseq analysis. Data are indicated as log2 cpm (counts per million). (**B**) Expression of Tcf1-dependent $CD8^+$ T cell stemness genes, identified in acute resolved infection, in WT and PD-1 KO T_{ML} and T_{EX} cells based on bulk RNAseq analysis. (**C**) Validation of the *Myb* shRNA constructs. In *vitro* activated $CD8^+$ T cells were transduced with Lentiviruses (LV) encoding *Myb*-sh RNA constructs. *Myb*-sh-expressing ($mCherry^+$) $CD8^+$ T cells were flow sorted and subjected to RT-qPCR analysis for *Myb*. Data in (**A, B**) derive from 2 biological replicates. Data in (**C**) represent n=3 technical replicates per condition. Bar graphs show means ±SD Statistics in (**A, B**): Unpaired two-tailed t-test between WT and PD-1 KO T_{ML} or T_{EX} cells. Statistics in (**C**) one-way ANOVA comparing the mean of each column to the scr sh control using uncorrected Fisher's LSD test. Significance (*$p<0.05$, **$p<0.01$, ***$p<0.001$, **** $p<0.0001$, ns=not significant $p>0.05$).

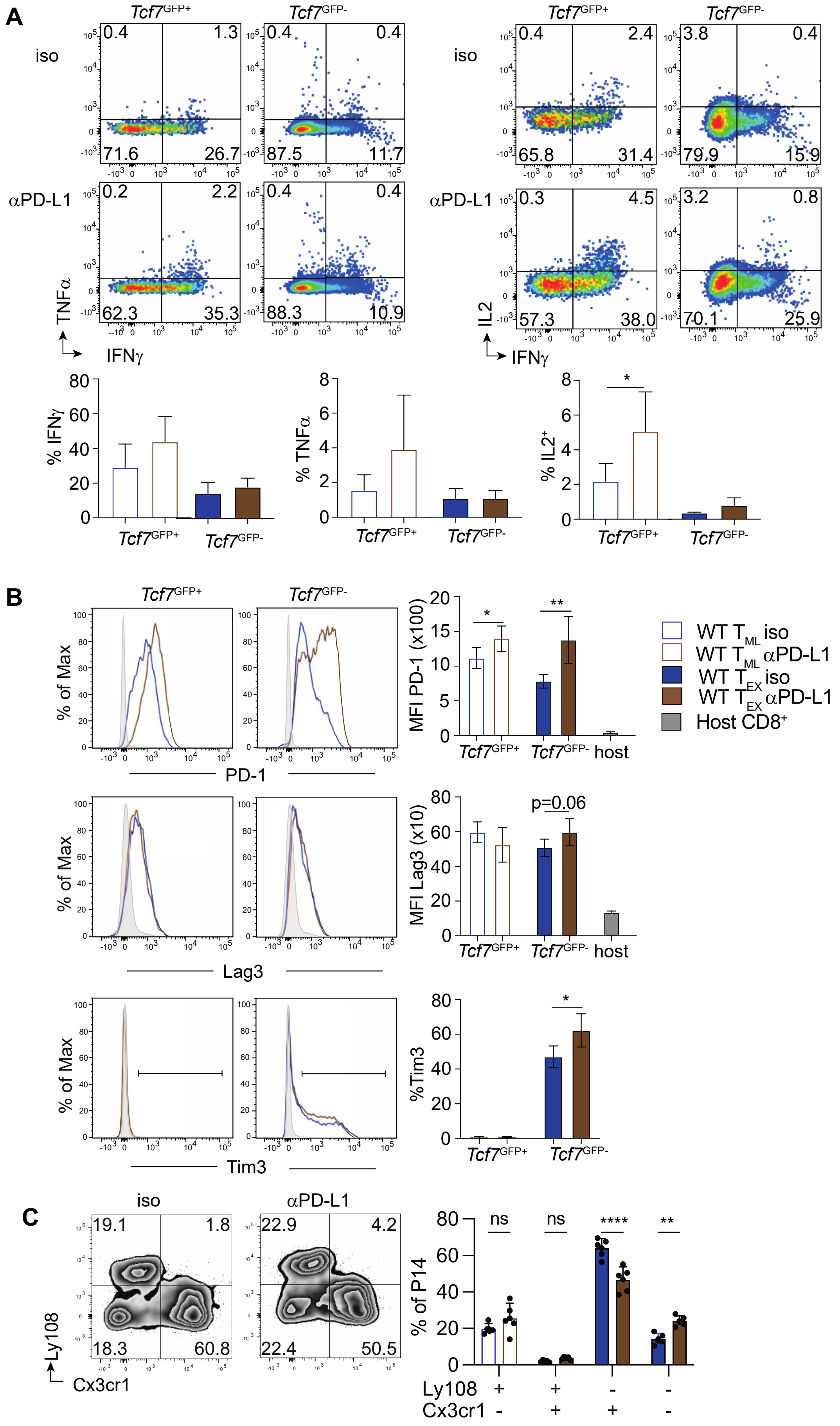
A
Tcf7GFP+
Tcf7GFP-
iso
αPD-L1
0.4
1.3
71.6
26.7
0.4
0.4
87.5
11.7
0.2
2.2
62.3
35.3
0.4
0.4
88.3
10.9
TNFα
IFNγ
0.4
2.4
65.8
31.4
3.8
0.4
79.9
15.9
0.3
4.5
57.3
38.0
3.2
0.8
70.1
25.9
IL2
% IFNγ
% TNFα
% IL2+
B
% of Max
PD-1
MFI PD-1 (x100)
Lag3
MFI Lag3 (x10)
p=0.06
Tim3
%Tim3
host
WT TML iso
WT TML αPD-L1
WT TEX iso
WT TEX αPD-L1
Host CD8+
C
iso
αPD-L1
19.1
1.8
18.3
60.8
22.9
4.2
22.4
50.5
Ly108
Cx3cr1
% of P14
ns
ns
****
**

Supplementary Figure 8: PD-L1 blockade reduces the self-renewal capacity of WT T_{ML} cells.
(**A, B**) WT *Tcf7*^{GFP} P14 cells (CD45.2) were injected into Vβ5 mice (CD45.1), which were then infected with LCMV cl13. Starting at d20 p.i., mice were treated with anti-PD-L1 (aPD-L1) or isotype control mAb, 5 times every 4th day. Mice were analyzed 5 days after the last injection on d41 p.i..
(**A**) Splenocytes at d41 p.i. were restimulated with gp33 peptide and gated T_{ML} (*Tcf7*$^{GFP+}$) and T_{EX} cells (*Tcf7*$^{GFP-}$) were analyzed for the production of IFNγ, TNFα and IL2. **(B)** Gated T_{ML} (*Tcf7*$^{GFP+}$) and T_{EX} cells (*Tcf7*$^{GFP+}$) from isotype- or aPD-L1-treated mice were analyzed for the expression of PD-1, Lag3 and Tim3 and compared to host CD8^{+} T cells (grey fill). (**C**) Mice were treated with aPD-L1 or isotype control mAb, 3 times every 3rd day. P14 cells were analyzed 3 days after the last injection (d33 p.i.) for the distribution of Ly108 versus Cx3cr1. Data in (**A, B**) derive from n=5-7 mice per group and are representative of n=3 independent experiments. Data in (**C**) derive from n=5 mice per group from a single experiment. Bar graphs show means (±SD). Statistics: Unpaired two-tailed t-test. Significance (*p<0.05, **p<0.01, ***p<0.001, **** p<0.0001, ns=not significant p>0.05).
